# Treatment utilisation for Aboriginal and Torres Strait Islander people diagnosed with prostate cancer in Australia: a population-based study

**DOI:** 10.1016/j.lanwpc.2026.101931

**Published:** 2026-08-04

**Authors:** Mei Ling Yap, Gabriel Gabriel, Joseph Descallar, Susan Anderson, Geoff P. Delaney, Viet Do, Rebecca Murray, Karen Wong, Henry H. Woo, Kalinda Griffiths, Keziah Bennett-Brook, Julieann Coombes

**Affiliations:** a The Cancer Program, The George Institute for Global Health, UNSW Sydney, Randwick, NSW, Australia; b Collaboration for Cancer Outcomes, Research and Evaluation (CCORE), Ingham Institute for Applied Medical Research, UNSW Sydney, Liverpool, NSW, Australia; c Liverpool and Macarthur Cancer Therapy Centres, Liverpool, NSW, Australia; d School of Medicine, Western Sydney University, Campbelltown, NSW, Australia; e South-Western Sydney Clinical Campus, School of Clinical Medicine, UNSW Sydney, Liverpool, NSW, Australia; f Ingham Institute for Applied Medical Research, Liverpool, NSW, Australia; g Aboriginal Consumer, Sydney, NSW, Australia; h Department of Urology, Blacktown & Mt Druitt Hospitals, Blacktown, NSW, Australia; i Department of Uro-oncology, Chris O’Brien Lifehouse, Camperdown, NSW, Australia; j Poche SA+NT, College of Medicine and Public Health, Flinders University, SA, Australia; k Centre for Big Data Research in Health, UNSW Sydney, Randwick, NSW, Australia; l Menzies School of Health Research, Tiwi, NT, Australia; m Guunu-maana (Heal) Aboriginal & Torres Strait Islander Health Program, The George Institute for Global Health, Randwick, NSW, Australia; n Yardhura Walani, National Centre for Epidemiology and Population Health, Australian National University, Canberra, Australia

**Keywords:** First Nations, Aboriginal and Torres Strait Islander people, Prostate cancer, Health equity, Patterns of care, Health disparities

## Abstract

**Background:**

Prostate cancer is the most common cancer diagnosed in Aboriginal and Torres Strait Islander men. Surgery and radiotherapy are curative options, with equivalent survival but different side effects and financial costs. We aim to determine the uptake of surgery and/or radiotherapy for prostate cancer by Aboriginal and Torres Strait Islander people within New South Wales (NSW).

**Methods:**

This was an individualised patient linked-data study which used the NSW Cancer Registry (2009–2018), NSW Admitted Patient Data Collection, Outpatient Radiation Oncology Data and Register of Births, Deaths and Marriages. Fine-Gray Competing Risk models were used to estimate cause-specific hazards for time to radiotherapy and time to prostatectomy with a competing risk of death.

**Findings:**

There were 59,621 people diagnosed with prostate cancer; 1061 (1.8%) were Aboriginal and Torres Strait Islander people. The median follow-up time was 6.3 years. A total of 22,946 (41.4%) patients received a prostatectomy; in Aboriginal and Torres Strait Islander people, it was lower at 362 (36.6%) patients. A total of 17,196 (31.0%) received external beam radiation therapy and for Aboriginal and Torres Strait Islander people, 349 (35.3%) received radiotherapy. In univariate analysis, Aboriginal and Torres Strait Islander people were less likely to receive a radical prostatectomy (HR 0.9, p < 0.05) than the rest of the population. However this association was lost when adjusting for possession of private insurance and comorbidities. Aboriginal and Torres Strait Islander people were more likely to receive radiotherapy on multivariable analysis (HR 1.2, p = 0.001).

**Interpretation:**

Aboriginal and Torres Strait Islander people are more likely to receive radiotherapy but less likely to receive surgery than the rest of the NSW population, with socio-economic factors associated with the treatment received. It is imperative there is equitable and affordable access to treatment options for Aboriginal and Torres Strait Islander people.

**Funding:**

This study was funded by the 2023 UNSW Health Systems Theme Collaborative grant.

**Research in context**

**Evidence before this study**

We searched Ovid and Pubmed for studies on Aboriginal and Torres Strait Islander people and prostate cancer, from inception until 30th December 2025 and then updated the search on 2nd June 2026. The search terms used were (“Aboriginal” or “Aboriginal and Torres Strait Islander” or “First Nation” or “Indigenous” or “Indigenous Australian” or “indigenous people” or “indigenous health care”) AND “Prostate cancer”. There are few studies focusing on prostate cancer in Aboriginal and Torres Strait Islander people. There was one population-based study in this area, published more than a decade ago. It found that Aboriginal men had less uptake of surgery for prostate cancer, but radiation therapy uptake was unable to be robustly studied due to a lack of data. This study also showed that Aboriginal men were more likely to die from prostate cancer than non-Indigenous patients.

**Added value of this study**

This is the largest Australian study using individualised population-based data to study prostate cancer in Aboriginal and Torres Strait Islander populations. It included 1061 Aboriginal and Torres Strait Islander people diagnosed with prostate cancer between 2009 and 2018 is also the first such work to study both the receipt of surgery and radiation therapy in these populations. We found that radiation therapy was received more often and surgery received less often, with possession of private health insurance and co-morbidities associated with the treatment received. We did not find differences in survival.

**Implications of all the available evidence**

This work highlights the need for affordable, publicly funded cancer care for Aboriginal and Torres Strait Islander people. It also reaffirms the need to prioritise the improvement of health outcomes more broadly for Aboriginal and Torres Strait Islander people, strengthening prevention, access to primary care and cultural safety in healthcare settings.

## Introduction

Aboriginal and Torres Strait Islander people are the First Peoples of Australia and have the longest continuing culture in the world.^1^ Centuries of colonisation have resulted in ongoing injustices experienced by Aboriginal and Torres Strait Islander people leading to historic and continuing health inequities.^2^ Within the Australian healthcare system, Aboriginal and Torres Strait Islander people continue to experience significant barriers to accessing services and are disproportionately affected by structural and systemic racism. These factors collectively contribute to persistent inequities in health outcomes and experiences of care.

Cancer is one of the most common causes of death in Aboriginal and Torres Strait Islander people.^3^ Prostate cancer is the most common cancer in Aboriginal and Torres Strait Islander men.^3^ Surgery and radiation therapy are curative treatment options which lead to equivalent survival but have different side effects^4^ and financial costs.^5^ Robotic-assisted laparoscopic prostatectomy is the most common curative surgical procedure for prostate cancer.^6^

As the most populous state in Australia, New South Wales (NSW) has a population of >8.4 million, with >278,000 Aboriginal and Torres Strait Islander people.^7^ There has been very little published data regarding prostate cancer in Aboriginal and Torres Strait Islander people. A prior study on Aboriginal and Torres Strait Islander people in NSW from 2001 to 2007 reported a low uptake of radical prostatectomies, however, the uptake of radiation therapy was unable to be robustly studied.^8^ This study concluded “*Larger and more detailed population-based studies of Australian Aboriginal men with prostate cancer are needed*”. As well, we have recently demonstrated that there are socioeconomic and sociodemographic drivers of the type of treatment received by people diagnosed with prostate cancer in wider NSW, including area level socioeconomic status, possession of private health insurance and the distance from the nearest treatment centre.^9^ The same associations have not been studied in the Aboriginal and Torres Strait Islander population.

In this study we aim to determine the factors associated with the utilisation of treatment—surgery and/or radiation therapy for prostate cancer in Aboriginal and Torres Strait Islander people. We will also examine the survival of Aboriginal and Torres Strait Islander people, compared to the rest of the NSW prostate cancer population and examine the factors associated with overall and cancer-specific mortality.

## Methods

### Study design and cohort selection

This was a retrospective population-based study using a large-scale linked dataset, consisting of routinely collected health and administrative data at an individualised patient level.

### Data source and linkage

The linked datasets included:

(1) NSW Cancer Registry (NSWCR; to 31st December 2018): notifications of primary cancer diagnosed in NSW residents;
(2) NSW Admitted Patient Data Collection (APDC; to December 2021): data on public and private hospital inpatient services;
(3) Outpatient Radiation Oncology Data (OROD; to December 2020) data on public and private radiation oncology outpatient treatments
(4) Register of Births, Deaths and Marriages (RBDM; to March 2021): all deaths that occurred in NSW
(5) Cause of death unit record file (CODURF): cause of all deaths that occurred in NSW; to December 2019

The timeframes of the APDC, OROD, RBDM and CODURF data were longer than the NSWCR due to both the recency of data availability of the different assets as well as the need for adequate follow up time to understand access to care and outcomes. Probabilistic linkage of records was performed by the NSW Centre for Health Record Linkage.^10^

### Aboriginal and Torres Strait Islander identification

We categorised patients as Aboriginal and/or Torres Strait Islander if any of the five linked databases identified the patient as Aboriginal and/or Torres Strait Islander, in keeping with previously reported methods.^11^ Those who declined or had “unknown” Aboriginal and Torres Strait Islander status were excluded from the study.

### Capturing prostate cancer diagnoses

All patients with a diagnosis of prostate cancer were identified from the NSWCR. Patients who were diagnosed based on death certificate only were excluded. The period of observation for each patient was the time from prostate cancer diagnosis to 31st March 2021.

### Receipt of treatment

For analyses regarding receipt of treatment, we excluded patients with metastatic disease on diagnosis. Patients were considered to have received radiotherapy if they had a code corresponding to external beam radiotherapy recorded on or after the diagnosis date in OROD and/or APDC records. Radiotherapy received from both public and private providers was captured using this method. Patients were considered to have received surgery if they had been captured as receiving a radical prostatectomy (which includes both open and robotic surgery) in the APDC. The relevant codes used to capture treatment are provided in Supplementary Table S1. For the analyses of radiation therapy or surgical utilisation, people whose nearest treatment facility was across the border were excluded, given their treatment might not be captured in NSW datasets. Patients diagnosed with >1 cancer were excluded as any radiotherapy received could not be attributed to a specific cancer diagnosis. Receipt of systemic therapy was not assessed due to incomplete data. While we could study patients who received neither radiation therapy nor surgery, we were unable to study those who were managed on an active surveillance programme.

### Other variables for analysis

Staging data were based on Degree of Spread data, the categorisation system used for the NSWCR. The 5 staging categories are: local, regional to adjacent organs, regional to lymph nodes, metastatic and unknown.

Patient place of residence was mapped to the Accessibility Remoteness Index of Australia.^12^ Area-level measure of socio-economic status was derived from the participant’s place of residence and classified into five quintiles using the Index of Relative Socioeconomic Disadvantage (IRSD).^13^ Road distances from patient residence to nearest radiotherapy facility and nearest robotic surgery facility was calculated through geocoding similar to previous methods,^14^ using Geographic Information System Software (ArcGIS Desktop: Release-10. Redlands, CA: Environmental Systems Research Institute).

Private health insurance possession was also an included variable. In Australia, all citizens and residents are covered by Medicare, the government-funded universal healthcare system, which covers all public inpatient costs and subsidises most outpatient health services. Additionally, people can elect to take private health insurance, providing additional reimbursement for out-of-pocket costs for some outpatient and inpatient services, including surgery undertaken at private facilities.

Non-cancer related co-morbidity scores were calculated, based on the Quan-modification of the Charlson Co-morbidity Index calculation (CCI),^15^ calculated using the ICD-10 diagnosis codes from the APDC dataset with a look back period of 2 years.

While data on the sex of each patient were available through the NSW cancer registry, gender information were not available.

### Study population and statistical analysis

To analyse receipt of treatments, Fine-Gray competing risk models were used to analyse each of radical prostatectomy receipt and external beam radiation receipt in separate models, both with a competing risk of death from any cause. This could include death from cancer or from other causes, including deaths related to medications such as Androgen deprivation therapy (ADT). Hierarchical modelling was used to investigate the effect of covariates on our outcomes of interest. Model 1 included Aboriginal status, age at diagnosis and socio-economic status. Model 2 added degree of spread, place of residence and year of diagnosis to model 1. Model 3 (the final model), then added relationship status, health insurance status, comorbidity, and distance to nearest treatment facility, and distance to robotics centre. Factors associated with cancer specific and overall mortality were analysed using Cox regression modelling. An interaction between degree of spread and receipt of radiation therapy was considered in the cancer specific and overall survival Cox models. In each of the Fine-Gray and Cox proportional hazards model, visual inspection of Schoenfeld residuals were used to assess proportional hazards assumption. To address violations of proportionality in the Fine-Gray models and Cox models, period-specific models were used to analyse sub-distribution hazard ratios and hazard ratios, respectively, over different time periods. Analyses were performed in SAS 9.4.

### Aboriginal and Torres Strait Islander leadership and ethics approval

The study was led by Aboriginal and Torres Strait Islander researchers, health workers and consumers (JC, KBB, KG, RM, SA) together with non-Indigenous clinicians and researchers, Central to the study governance was an Aboriginal and Torres Strait Islander Steering Committee, consisting of researchers (including expertise in Indigenous data sovereignty, Indigenous research methodologies and decolonising traditional methdologies), hospital and community health workers and Aboriginal people with lived experience. The research topic was identified as a priority by the Steering Committee as well as the wider Aboriginal health service and community. The study gained research ethics approval from the NSW Population and Health Services Research Ethics Committee (NSW Ethics 2019/ETH01657) and the Aboriginal Health and Medical Research Ethics Committee (NSW AH&MRC 1770/21).

### Role of the funding source

This study was funded by the 2023 UNSW Health Systems Theme Collaborative grant. The funder had no role in the study design, data collection, data analysis, interpretation of data or writing of the report.

## Results

After record linkage and exclusions, there were 59,621 people diagnosed with prostate cancer during our study between the years 2009–2018; 1061 (1.8%) of those were Aboriginal and/or Torres Strait Islander people. There were 358 patients who either declined or had unknown Aboriginal or Torres Strait Islander status. A total of 4191 patients had a nearest treatment facility across state borders, 72 of those were Aboriginal and Torres Strait Islander people. The median age was 67 years (IQR 61–74 years) and the median follow up time since diagnosis was 6.3 years (IQR 3.8–9.0 years). All patients were of male sex.

The baseline characteristics are featured in Table 1. Aboriginal and Torres Strait Islander patients were younger at diagnosis compared to non-Indigenous patients (26% versus 20% aged <60, median age was 65 versus 67), were more likely to live in moderately accessible or remote communities (36 versus 19%) and more than 200 km from the nearest treatment centre (13 versus 5%, median distance = 26 versus 9 km). Non-Indigenous patients were more likely to hold private health insurance, live in advantaged socio-economic status suburbs and be married.

**Table 1:** Baseline characteristics.

Characteristic	Aboriginal and/or Torres Strait Islander	Non-indigenous	p value
	N%	N%	
Year of diagnosis			
2009–2013	510 (48%)	29,791 (51%)	0.304
2014–2018	551 (52%)	28,769 (49%)	
Age			
<60 years	279 (26%)	11,613 (20%)	<0.001
60–69 years	456 (43%)	23,133 (40%)	
70–79 years	262 (25%)	17,037 (29%)	
80+ years	64 (6%)	6777 (12%)	
Stage			
Localised	570 (54%)	30,330 (52%)	0.001
Regional	118 (11%)	9298 (16%)	
Distant	49 (5%)	2604 (4%)	
Unknown	324 (30%)	16,328 (28%)	
Index of relative socioeconomic disadvantage			
IRSD-1 (Most disadvantaged)	291 (27%)	10,499 (18%)	<0.001
IRSD-2	260 (25%)	11,226 (19%)	
IRSD-3	211 (20%)	11,169 (19%)	
IRSD-4	180 (17%)	12,371 (21%)	
IRSD-5 (Least disadvantaged)	113 (11%)	13,249 (23%)	
Unknown	X^a^	46 (0%)	
Accessibility/remoteness index of area (ARIA+)			
Highly accessible	406 (38%)	32,810 (56%)	<0.001
Accessible	277 (26%)	14,680 (25%)	
Moderately accessible	336 (32%)	10,549 (18%)	
Remote	42 (4%)	509 (1%)	
Unknown	0 (0%)	12 (0%)	
Distance to nearest radiotherapy facility			
<50 km	644 (61%)	46,026 (78%)	<0.001
50–99 km	144 (14%)	5054 (9%)	
100–149 km	87 (8%)	3039 (5%)	
150–199 km	47 (4%)	1576 (3%)	
200+ km	139 (13%)	2857 (5%)	
Quan comorbidity score			
Score 0	709 (67%)	43,731 (75%)	<0.001
Score 1	151 (14%)	5661 (10%)	
Score 2+	201 (19%)	9168 (15%)	
Marital status			
Married	800 (75%)	48,253 (82%)	<0.001
Single	250 (24%)	9328 (16%)	
Unknown	11 (1%)	979 (2%)	
Holds private health insurance			
Yes	510 (48%)	39,829 (68%)	<0.001
No	538 (51%)	17,504 (30%)	
Unknown	13 (1%)	1227 (2%)	

a Number suppressed due to data privacy reasons.

### Treatments received

**Fig. 1:**
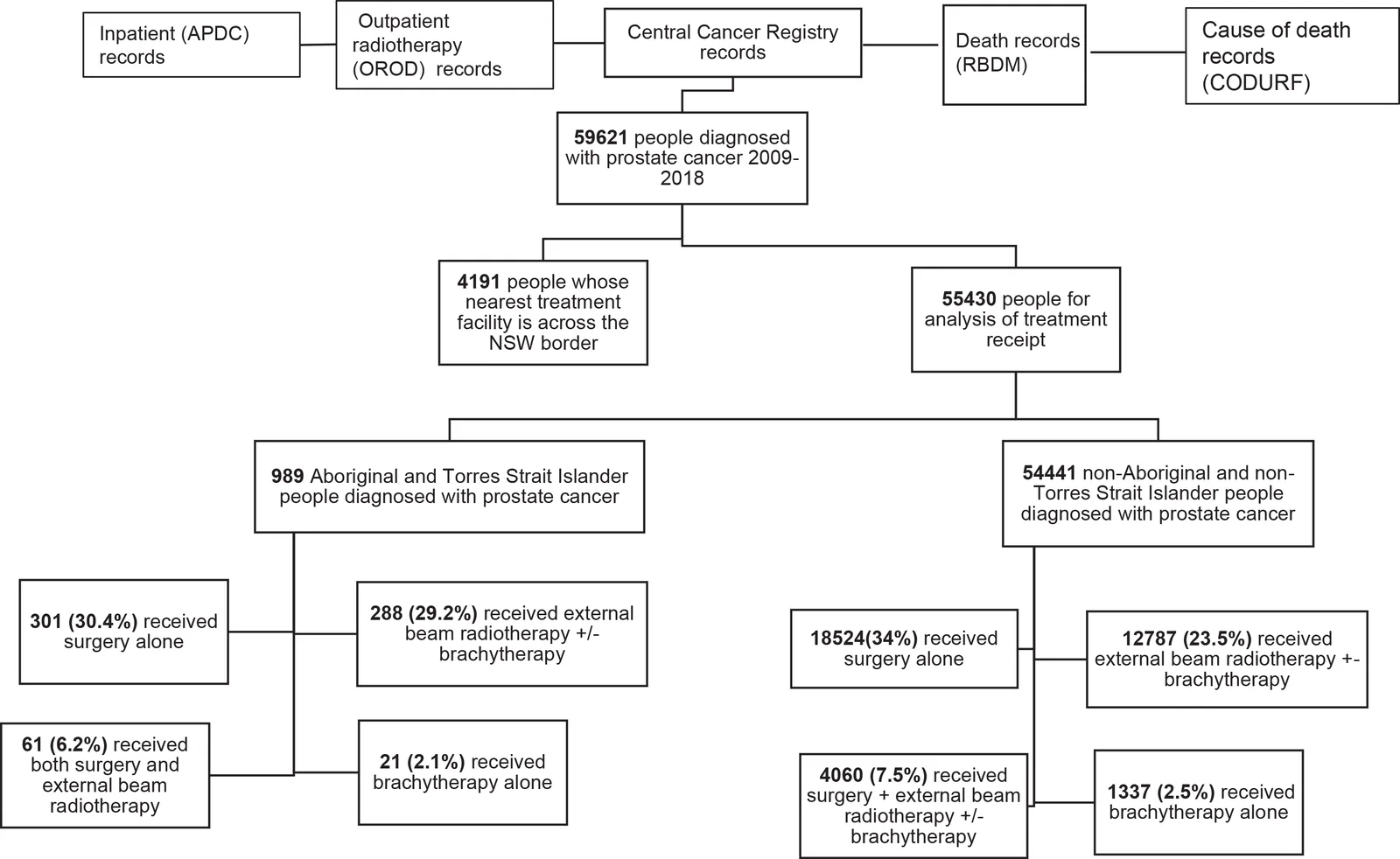
Treatments received.

The treatments received are outlined in Fig. 1. A total of 22,946 (41.4%) of patients received a radical prostatectomy, the rate was higher in non-Indigenous patients (22,584, 41.5%) compared to Aboriginal and Torres Strait Islander patients (362, 36.6%) p = 0.002. Of those who received a radical prostatectomy, 76% had these performed in private hospitals, however the rates differed between non- Indigenous (76%) compared to Aboriginal and Torres Strait Islander patients (55%). A total of 17,196 (31%) of patients received external beam radiation therapy (EBRT), the rate was higher in Aboriginal and Torres Strait Islander patients (349, 35.3%), compared to 16,847 in non-Indigenous patients (30.9%) (p = 0.002). There were 2146 patients (3.6%) who received brachytherapy, 2118 (3.6%) were in non-Indigenous patients and 28 (2.6%) in Aboriginal and Torres Strait Islander patients. Of those patients who received both surgery and EBRT (N = 4090), 1027 (25%) patients received this in the adjuvant setting, while the remainder received it in on relapse, in either the salvage or palliative setting. Of Aboriginal and Torres Strait Islander people who received both treatments (N = 61), 14 (23%) received it in the adjuvant setting. The proportion of patients who did not receive surgery or radiotherapy was similar, 32.3% in Aboriginal patients and 32.6% in non-Indigenous patients.

### Factors associated with type of treatment received

Univariate analyses and multivariable regression analyses for patients with non-metastatic prostate cancer undergoing radical prostatectomy or EBRT, adjusted for age and stage of disease, are shown in Tables 2 and 3. In the univariable analysis, being Aboriginal and Torres Strait Islander was associated with lower surgical utilisation and higher radiotherapy utilisation. However, when models were adjusted for private health insurance possession and co-morbidities in a step-wise fashion, being Aboriginal and Torres Strait Islander was no longer significant for surgical utilisation.

**Table 2:** Subdistribution model for surgery receipt with competing risk of death.^a^

Sociodemographic or health factor	Univariate model		Multivariable model without insurance and comorbidities		Final multivariable model	
	SHR (95% CI)	p	SHR (95% CI)	p	SHR (95% CI)	p
Aboriginal and Torres Strait Islander identification		<0.001		<0.001		0.071
Aboriginal and/or Torres Strait Islander	0.82 (0.74, 0.91)		0.81 (0.72, 0.90)		0.91 (0.81, 1.01)	
Non-indigenous	Reference		Reference		Reference	
Age at diagnosis (years)		<0.001		<0.001		<0.001
<60	Reference		Reference		Reference	
60–69	0.81 (0.79, 0.84)		0.79 (0.77, 0.82)		0.82 (0.79, 0.84)	
70–79	0.32 (0.31, 0.34)		0.33 (0.31, 0.34)		0.36 (0.35, 0.38)	
80 or more	0.02 (0.02, 0.02)		0.02 (0.02, 0.02)		0.02 (0.02, 0.03)	
Place of residence		<0.001		0.073		0.063
Major cities	Reference		Reference		Reference	
Inner regional	0.93 (0.90, 0.96)		1.04 (1.00, 1.08)		1.04 (1.01,1.08)	
Outer regional/remote/very remote	0.89 (0.86, 0.92)		1.00 (0.91, 1.09)		1.01 (0.92, 1.10)	
Index of relative socioeconomic disadvantage		<0.001		<0.001		<0.001
IRSD-1 (Most disadvantaged)	Reference		Reference		Reference	
IRSD-2	1.15 (1.09, 1.20)		1.11 (1.05, 1.16)		1.06 (1.01, 1.12)	
IRSD-3	1.18 (1.12, 1.23)		1.18 (1.13, 1.24)		1.11 (1.05, 1.16)	
IRSD-4	1.36 (1.31, 1.43)		1.31 (1.25, 1.38)		1.19 (1.13, 1.25)	
IRSD-5 (Least disadvantaged)	1.66 (1.60, 1.73)		1.56 (1.49, 1.64)		1.34 (1.28, 1.41)	
Year of diagnosis		<0.001		<0.001		<0.001
2014–2018	Reference					
2009–2013	1.06 (1.03, 1.09)		1.06 (1.03, 1.09)		1.08 (1.05,1.11)	
Degree of spread		<0.001		<0.001		<0.001
Localised	Reference		Reference		Reference	
Regionalised, adjacent organs	3.21 (3.11, 3.32)		3.25 (3.14, 3.36)		3.20 (3.09, 3.32)	
Regionalised, lymph nodes	3.02 (2.75, 3.31)		3.06 (2.78, 3.36)		3.17 (2.89, 3.48)	
Unknown	0.36 (0.35, 0.37)		0.40 (0.38, 0.41)		0.40 (0.38, 0.41)	
Relationship status		<0.001		<0.001		<0.001
Married/partnered	Reference		Reference		Reference	
Single	0.62 (0.60, 0.65)		0.66 (0.63, 0.68)		0.74 (0.71, 0.77)	
Missing data	0.19 (0.14, 0.25)		0.16 (0.12, 0.21)		0.20 (0.14, 0.27)	
Distance from nearest radiotherapy facility		<0.001		<0.001		<0.001
<50 km	Reference		Reference		Reference	
50–99 km	0.78 (0.74, 0.53)		1.09 (1.00, 1.19)		1.08 (0.99, 1.17)	
100–149 km	0.85 (0.79, 0.91)		1.33 (1.21, 1.47)		1.35 (1.22, 1.49)	
150–199 km	1.05 (0.97, 1.14)		1.45 (1.29, 1.63)		1.46 (1.29, 1.65)	
200+ km	1.44 (1.36, 1.53)		2.26 (2.04, 2.50)		2.30 (2.07, 2.55)	
Distance from nearest robotic surgery centre		<0.001		<0.001		<0.001
<50 km	Reference		Reference		Reference	
50–99 km	0.79 (0.74, 0.84)		0.82 (0.74, 0.89)		0.80 (0.73, 0.88)	
100–149 km	0.79 (0.74, 0.85)		0.75 (0.67, 0.83)		0.73 (0.66, 0.81)	
150–199 km	0.72 (0.66, 0.77)		0.79 (0.72, 0.87)		0.78 (0.71, 0.86)	
200+ km	0.78 (0.74, 0.82)		0.76 (0.71, 0.81)		0.74 (0.69, 0.79)	
Private insurance		<0.001				<0.001
Yes	Reference				Reference	
No	0.54 (0.52, 0.55)		–	–	0.66 (0.64, 0.69)	
Unknown/missing	0.41 (0.35, 0.47)				0.60 (0.50, 0.72)	
Charlson comorbidity index (Quan)		<0.001				<0.001
0	Reference		–	–	Reference	
1	0.63 (0.60, 0.66)				0.81 (0.77, 0.85)	
2 or more	0.32 (0.31, 0.34)				0.57 (0.54, 0.60)	

a Includes patients with non-metastatic disease only. Fine-Gray competing risk models were used, with a competing risk of death from any cause. Hierarchical modelling was used to investigate the effect of covariates on our outcomes of interest.

**Table 3:** Subdistribution model for radiation therapy receipt with competing risk of death.^a^

Sociodemographic or health factor	Univariate model		Multivariable model without insurance and comorbidities		Final multivariable model	
	SHR (95% CI)	p	SHR (95% CI)	p	SHR (95% CI)	p
Aboriginal and Torres Strait Islander identification		0.001		<0.001		<0.001
Aboriginal and/or Torres Strait Islander	1.20 (1.07, 1.34)		1.29 (1.15, 1.44)		1.22 (1.09, 1.36)	
Non–indigenous	Reference		Reference		Reference	
Age at diagnosis (years)		<0.001		<0.001		<0.001
<60	Reference		Reference		Reference	
60–69	1.68 (1.60, 1.77)		1.65 (1.57, 1.69)		1.65 (1.57, 1.73)	
70–79	2.89 (2.75, 3.03)		2.82 (2.68, 2.97)		2.76 (2.63, 2.91)	
80 or more	1.55 (1.44, 1.66)		1.51 (1.41, 1.62)		1.47 (1.36, 1.58)	
Place of residence		<0.001		<0.001		<0.001
Major cities	Reference		Reference		Reference	
Inner regional	1.13 (1.09, 1.18)		1.10 (1.05, 1.15)		1.10 (1.05, 1.15)	
Outer regional/remote/very remote	1.05 (1.01, 1.10)		1.43 (1.31, 1.56)		1.43 (1.31, 1.57)	
Index of relative socioeconomic disadvantage		<0.001		<0.001		<0.001
IRSD-1 (Most disadvantaged)	Reference		Reference		Reference	
IRSD-2	0.91 (0.86, 0.95)		0.91 (0.87, 0.96)		0.94 (0.89, 0.99)	
IRSD-3	0.96 (0.91, 1.00)		0.93 (0.88, 0.98)		0.98 (0.93, 1.03)	
IRSD-4	0.84 (0.80, 0.88)		0.81 (0.77, 0.85)		0.88 (0.83, 0.92)	
IRSD-5 (Least disadvantaged)	0.71 (0.67, 0.74)		0.69 (0.66, 0.73)		0.78 (0.74, 0.82)	
Year of diagnosis		<0.001		<0.001		<0.001
2014–2018	Reference		Reference		Reference	
2009–2013	0.81 (0.79, 0.84)		0.87 (0.84,0.90)		0.87 (0.84,0.89)	
Degree of spread		<0.001		<0.001		<0.001
Localised	Reference		Reference		Reference	
Regionalised, adjacent organs	1.36 (1.31, 1.42)		1.36 (1.30, 1.42)		1.39 (1.34, 1.46)	
Regionalised, lymph nodes	2.25 (2.07, 2.46)		2.18 (1.98, 2.39)		2.18 (1.98, 2.39)	
Unknown	1.27 (1.23, 1.32)		1.18 (1.14, 1.22)		1.16 (1.12, 1.21)	
Relationship status		<0.001		<0.001		<0.001
Married/partnered	Reference		Reference		Reference	
Single	1.10 (1.06, 1.15)		1.10 (1.05, 1.15)		1.01 (0.97, 1.06)	
Missing data	1.32 (1.13, 1.54)		1.35 (1.15, 1.58)		1.56 (1.31, 1.86)	
Distance from nearest treatment facility		<0.001		<0.001		<0.001
<50 km	Reference		Reference		Reference	
50–99 km	1.18 (1.12, 1.24)		0.76 (0.69, 0.82)		0.76 (0.69, 0.82)	
100–149 km	1.02 (0.95, 1.10)		0.63 (0.57, 0.70)		0.63 (0.57, 0.70)	
150–199 km	0.76 (0.68, 0.85)		0.47 (0.41, 0.54)		0.46 (0.40, 0.53)	
200+ km	0.46 (0.41, 0.51)		0.26 (0.23, 0.30)		0.25 (0.22, 0.29)	
Distance from nearest robotic surgery facility		<0.001		0.164		0.066
<50 km	Reference		Reference		Reference	
50–99 km	1.12 (1.05, 1.20)		1.12 (1.02, 1.24)		1.15 (1.04, 1.26)	
100–149 km	1.14 (1.05, 1.22)		1.06 (0.95, 1.18)		1.07 (0.96, 1.18)	
150–199 km	1.21 (1.12, 1.30)		1.04 (0.94, 1.14)		1.04 (0.95, 1.15)	
200+ km	1.07 (1.02, 1.13)		1.01 (0.94, 1.08)		1.01 (0.94, 1.09)	
Health insurance status		<0.001				<0.001
Private health insurance	Reference				Reference	
No private insurance	1.45 (1.40, 1.50)				1.39 (1.34, 1.44)	
Unknown/missing	1.11 (0.97, 1.28)				0.92 (0.79, 1.08)	
Charlson comorbidity index (Quan)		<0.001				<0.001
0	Reference				Reference	
1	1.30 (1.24, 1.36)				1.13 (1.08, 1.19)	
2 or more	1.17 (1.12, 1.22)				1.00 (0.95, 1.05)	

a Includes patients with non-metastatic disease only Fine-Gray competing risk models were used, with a competing risk of death from any cause. Hierarchical modelling was used to investigate the effect of covariates on our outcomes of interest.

From the 59,621 people diagnosed with prostate cancer during the study period, 55,283 were used in the Fine-Gray and Cox regression models. In total, 4338 patients were excluded due to cross-border patients (n = 4191), negative survival times (n = 99) or IRSD being unknown (n = 52). Given the small number of patients with unknown data, multiple imputation was not performed.

In the multivariable analysis, factors associated with greater likelihood of radical prostatectomy were: being younger age, living in a less disadvantaged city, being diagnosed in 2009–2013, having regionalised disease, being married/partnered, living further from the nearest radiation therapy centre, living closer to the nearest robotic surgical centre, owning private health insurance and not having co-morbidities.

Factors associated with greater likelihood of EBRT were: being Aboriginal and Torres Strait Islander, being aged 70–79, living in outer regional/remote areas, being most socio-economically disadvantaged, being diagnosed in 2014–2018, having regionalised, distant or unknown disease stage, living closer to the nearest radiotherapy facility, not holding private health insurance and having one comorbidity.

For each of the radical prostatectomy and EBRT models, evidence of non-proportionality was observed for most variables. Treatment decisions are most often considered within the first 12 months of diagnosis. The period-specific analyses for these models indicate that by 12 months, the sub-distribution hazard ratios are in large agreement with the main analyses. This is expanded on in the supplementary material (Supplementary Table S2).

### Survival

For all patients, 5-year overall survival (OS) was 87.5%, 87.8% for Aboriginal and Torres Strait Islander patients and 87.5% for non-Indigenous patients (p = 0.217). The 5-year cancer-specific survival was 93.5%, 93.3% for Aboriginal and Torres Strait Islander patients and 93.5% for non-Indigenous patients (p = 0.528). When adjusting for socio-demographic, socio-economic and treatment factors, being Aboriginal and Torres Strait Islander was not associated with worse overall or cancer-specific survival (Table 4). Since proportional hazards assumption was not satisfied based on Schoenfeld residuals, we performed a time varying analysis using period-specific models (Supplementary Tables S3 and S4). This showed that the association of radiotherapy receipt and worsened survival was only in the >12 months post diagnosis timeframe (whereas radiotherapy was associated with better survival ≤12 months timeframe), likely due to the typical indication of radiotherapy when given within 12 months (curative intent) versus >12 months post diagnosis (palliative intent).

**Table 4:** Cox regression analysis on overall and cancer specific mortality.

Sociodemographic or health factor	Overall mortality		Cancer specific mortality (intermediate model)	
	Hazard (95% CI)	p value	Hazard (95% CI)	p value
Aboriginal and Torres Strait Islander identification				
Non-Aboriginal and Torres Strait Islander	Reference			
Aboriginal and/or Torres Strait Islander	1.13 (0.98, 1.31)	0.104	1.16 (0.92,1.45)	0.217
Age group				
<60 years	Reference		Reference	
60–69 years	1.5	<0.001	1.21 (1.05, 1.40)	0.007
70–79 years	2.88 (2.61, 3.17)	<0.001	1.88 (1.64, 2.16)	<0.001
80+ years	7.49 (6.77, 8.28)	<0.001	5.46 (4.75, 6.27)	<0.001
Year of diagnosis				
2014–2018	Reference			
2009–2013	1.03 (0.99, 1.08)	0.163	1.38 (1.29, 1.48)	<0.001
Index of relative socioeconomic disadvantage				
IRSD-1 (Most disadvantaged)	Reference			
IRSD-2	1.02 (0.96, 1.09)	0.504	1.11 (1.01, 1.22)	0.037
IRSD-3	0.98 (0.92. 1.05)	0.568	0.98 (0.88, 1.08)	0.632
IRSD-4	1.00 (0.93. 1.07)	0.939	1.01 (0.91, 1.13)	0.797
IRSD-5 (Least disadvantaged)	0.95 (0.89. 1.02)	0.163	1.03 (0.92, 1.15)	0.585
Degree of spread				
Localised to tissue of origin	Reference		Reference	
Regional spread, adjacent organs	1.89 (1.75, 2.04)	<0.001	3.12 (2.75, 3.53)	<0.001
Regional spread, regional LN	4.32 (3.72, 5.03)	<0.001	8.28 (6.74, 10.17)	<0.001
Distant metastasis	7.75 (7.30, 8.22)	<0.001	18.69 (17.14, 20.38)	<0.001
Unknown	1.22 (1.17, 1.29)	<0.001	1.58 (1.45, 1.73)	<0.001
Accessibility/remoteness index of Australia (ARIA+)				
Major cities	Reference		Reference	
Inner regional	1.12 (1.06, 1.19)	<0.001	1.24 (1.13, 1.35)	<0.001
Outer regional/Remote/very remote	1.07 (0.96, 1.20)	0.221	1.14 (0.97, 1.35)	0.118
Distance from RT centre				
<50 km	Reference		Reference	
50–99 km	1.15 (1.03, 1.28)	0.015	1.15 (0.98, 1.36)	0.091
100–149 km	1.13 (0.99, 1.28)	0.075	1.05 (0.86, 1.28)	0.639
150–199 km	1.15 (0.97, 1.36)	0.107	1.21 (0.94, 1.55)	0.147
200+ km	1.45 (1.27, 1.67)	<0.001	1.43 (1.16, 1.76)	0.001
Quan comorbidity index (QCI)				
No comorbidity	Reference		Reference	
QCI = 1	1.59 (1.49, 1.7)	<0.001	1.4 (1.28, 1.54)	<0.001
QCI ≥ 2	2.82 (2.69, 2.96)	<0.001	1.5 (1.39, 1.61)	<0.001
Marital status				
Married	Reference		Reference	
Single	1.33 (1.27, 1.39)	0.001	1.28 (1.19, 1.38)	<0.001
Missing	0.84 (0.58, 1.21)	0.337	0.56 (0.31, 0.99)	0.046
Private insurance				
Had private insurance	Reference		Reference	
Did not have private insurance	1.29 (1.23, 1.35)	<0.001	1.28 (1.19, 1.37)	<0.001
Missing/Unknown	0.90 (0.67, 1.21)	0.484	0.85 (0.55, 1.31)	0.457
Radiation therapy				
Not received	Reference		Reference	
Received	1.03 (0.99, 1.07)	0.205	1.49 (1.40, 1.59)	<0.001
Radical prostatectomy				
Not received	Reference		Reference	
Received	0.29 (0.27, 0.32)	<0.001	0.17 (0.15, 0.2)	<0.001
Distance to robotic centre				
<50 km	Reference		Reference	
50–99 km	1.03 (0.92, 1.16)	0.623	1.03 (0.86, 1.24)	0.742
100–149 km	0.98 (0.86, 1.12)	0.790	1.02 (0.83, 1.25)	0.857
150–199 km	0.98 (0.87, 1.11)	0.767	0.95 (0.79, 1.15)	0.619
200+ km	0.86 (0.79, 0.95)	0.002	0.88 (0.76, 1.00)	0.057

Diagnostic plots for both the Fine Gray and Cox models are found in the Supplementary (Supplementary Figs.).

## Discussion

This is the largest Australian study using individualised population-based data to study the patterns of care for prostate cancer in Aboriginal and Torres Strait Islander populations. It is also the first to study both the receipt of surgery and radiation therapy in Aboriginal and Torres Strait Islander people diagnosed with prostate cancer.

A prior study from New South Wales in 2007–2011, found that Aboriginal patients were 40% less likely to receive surgery than non-Indigenous patients,^8^ and were more likely to die from prostate cancer than non-Indigenous patients. In our study, we did not find the same survival differences although the finding of reduced uptake of surgery persisted. The improvements in survival found may relate to better diagnostic pathways, active surveillance protocols and/or higher utilisation of treatment over time.^16^

Our study found that Aboriginal and Torres Strait Islander men received surgery less often than non-Indigenous men. However, when adjusting for private health insurance possession and comorbidities, being Aboriginal and Torres Strait Islander had no association with receipt of surgery in the multivariable model. We also found that when surgery was received, just under half of Aboriginal and Torres Strait Islander people received surgery in the public system, whereas within the overall population, 75% of people received surgery in the private system.^17^ Rates of possession of private health insurance in Australia are known to be lower in Aboriginal and Torres Strait Islander populations.^18^ A recent meta-analysis found a lower receipt of cancer surgery by Aboriginal and Torres Strait Islander people.^19^ This study also highlighted a specific barrier for NSW–that there are fewer surgical outpatient clinics compared to other Australian states, resulting in the need for patients to access the private sector to receive care. Out of pocket costs for prostatectomies in NSW are the highest in Australia, typically $9200 AUD per patient.^20^ Ensuring the provision of publicly available surgical services to meet the needs of people with prostate cancer within NSW, regardless of socio-economic background, is imperative. There has been growing focus on the need to prevent financial toxicity in prostate cancer care within the Australian setting.^5^

Our study also found that Aboriginal and Torres Strait Islander men were more likely to receive radiation therapy. We postulate that this may in part be driven by higher risk prostate cancer features such as higher PSA or Gleason score, which may be related to later diagnosis of prostate cancer in Aboriginal and Torres Strait Islander men. However, the receipt of radiotherapy was also associated with being from a more disadvantaged area, living in outer regional/remote areas and not possessing private health insurance; factors which may relate to these populations being less likely to undertake surgery.

Structural factors within the Australian health system, including the centralisation of some specialised oncological treatment modalities, may influence the care received for prostate cancer for Aboriginal and Torres Strait Islander people. Our study found that Aboriginal and Torres Strait Islander people are more likely to live in remote areas where there may be limited access to specialised cancer care. The changing nature of cancer service provision (public versus private) within the Australian health system is another consideration which can affect treatment access for Aboriginal and Torres Strait Islander people. Radiation therapy is predominantly delivered in the public system, with around two-thirds of care in 2018–2019 occurring in public centres.^21^ Radiation therapy delivered in the public sector is typically associated with little or no out of pocket costs for the treatment itself. However, in recent years, there has been growth in the number of privately-owned radiotherapy centres. While some of these centres are public-private partnerships which have allowed for rural communities to access much-needed care free of charge, many patients receiving radiotherapy in the private sector incur significant out-of-pocket costs.^22^ It is imperative that publicly available radiation therapy, either through public sector or public-private partnerships, remain available to all men with prostate cancer in NSW, including Aboriginal and Torres Strait Islander people.

Having comorbidities was associated with reduced surgical receipt for Aboriginal and Torres Strait Islander people and was associated with increased radiotherapy receipt. A prior study from Queensland, Australia, across all cancer types, highlighted that Indigenous people diagnosed with cancer had more comorbidities compared to non-Indigenous people.^23^ It also found that Indigenous people had lower receipt of curative treatment than non-Indigenous people, but that this was true for people with and without a comorbidity. These findings highlight the issue of cancer within the setting of wider multi-morbidity and the need to continue to prioritise the improvement of health outcomes more generally for Aboriginal and Torres Strait Islander people, strengthening prevention, access to primary care^18^ and cultural safety in healthcare settings.

The finding of lower surgical utilisation and higher radiotherapy utilisation in Aboriginal and Torres Strait Islander people is similar to the findings in recent population-based studies in both US American Indian and Alaskan Native populations^24^ and Canadian First Nations prostate cancer populations,^25^ even though the health systems differ. Although the Aboriginal and Torres Strait Islander populations of Australia are very much unique with respect to culture, history and language, there are some similar themes shared in the history of colonisation, oppression and trauma. While differences in cancer stage and risk grouping may in part play a role, the fragmentation of the Australian health system and the lack of cultural safety in a health system rooted in colonisation, may also contribute to the current care pathways for Aboriginal and Torres Strait Islander people.

Additionally, referral pathways play an important role in treatment access. In Australia, the diagnosis of prostate cancer is typically made by a urologist. It has been previously demonstrated in NSW that only 1 in 8 men with prostate cancer had a consultation with a radiation oncologist prior to undergoing prostatectomy, raising the issue of patients not being offered an equally effective alternative.^9^ In light of this issue, changes to the Medicare Benefits Schedules for radical prostectomy items were made in 2020, stipulating multidisciplinary team review and a discussion regarding referral to a radiation oncologist, prior to surgery.^26^

To address the issue of structural racism and a lack of cultural safety in the health system, capacity building to include Aboriginal and Torres Strait Islander people within the cancer care workforce is paramount. Recent models have included the provision of an Aboriginal and Torres Strait Islander patient navigator to ensure the journey through the cancer health system is more culturally safe.^27^ However, the oncology workforce in Australia still severely lacks Aboriginal and Torres Strait Islander representation. While there are current measures to try and build the Aboriginal and Torres Strait workforce in oncology, such as the Royal and New Zealand College of Radiologists’ Indigenous scholarships, this is a long-term endeavour. In the shorter term, approaches to improve cultural understanding of the non-Indigenous workforce is much needed. Our research group’s next endeavour is an Aboriginal and Torres Strait led cultural safety training course for oncology health care providers, adapted from recent successes in the setting of burns management in children.^28^

Patient preference in the setting of prostate cancer treatment is an important consideration, which we were not able to account for in the study. Surgery and radiotherapy are both effective treatments for prostate cancer, but are very different with regards to invasiveness, side effect profiles and quality of life implications. While surgery is associated with higher rates of urinary incontinence and sexual dysfunction, radiotherapy is associated with higher rates of bowel toxicity.^4^ Additionally, some patients who undergo radiotherapy may experience side effects from the use of concombinant ADT.^29^ Cultural determinants may also shape decision making around treatment, with expectations and trust previously highlighted as important factors in the surgical care journey.^30^ Further qualitative research regarding the additional reasons for the lower uptake of surgery and higher uptake of radiotherapy in Aboriginal and Torres Strait Islander people diagnosed with prostate cancer is needed.

The strengths of this study include the use of a large-scale population-based dataset and the leadership of Aboriginal and Torres Strait Islander researchers, health workers and consumers within the research team. The weaknesses of this study pertain to the limitations of the dataset, including the lack of more granular prostate cancer risk factors (PSA, Gleason score) and clinical factors such as urinary score and performance status. We also could only report on summary stage due to the data available in the cancer registry, which lacked the more granular TNM staging. It has previously been demonstrated that American Indian and Alaskan Native peoples in the United States of America had higher Gleason score and higher risk prostate cancer than other racial groups,^24^ but we were unable to study this due to the lack of data. The finding of a lower proportion of regionalised disease in Aboriginal and Torres Strait Islander people may relate in part to lower surgery rates (and therefore less lymph node sampling) but it may also be related to differences in PSMA PET access—however data on diagnostic imaging were not available. We also found a relatively high proportion of patients with “unknown stage” and a lack of data on the use of systemic therapies. We also could not identify patients who were on active surveillance. Although we were able to access information from multiple population-based datasets, the longstanding issue of data completeness and underidentification of Aboriginal and Torres Strait people within health and administrative datasets remains.^31^ As well, this study only includes patients from New South Wales and the findings may not be applicable to other states and territories within Australia, given the diversity of Aboriginal and Torres Strait Islander Nations and communities, geography and health systems. The recent availability of national cancer data^32^ may allow for future work to better understand whether these results are widely applicable to Aboriginal and Torres Strait Islander populations outside of NSW.

### Conclusion

In the largest population-based study of Aboriginal and Torres Strait Islander men diagnosed with prostate cancer, we found that there was lower uptake of surgery and higher uptake of radiotherapy and this was associated with the limited possession of private health insurance and prevalence of comorbidities in Aboriginal and Torres Strait Islander people. This highlights the need for policy and health services planning to ensure equitable access for Aboriginal and Torres Strait people to publicly funded cancer care and healthcare more broadly.

## Contributors

Conceptualisation–MLY, JC, KBB, GPD, GG, KG.

Data curation–GG, JD.

Formal Analysis–GG, JD.

Funding Acquisition–MLY, JC, GG, SA, GPD.

Investigation–MLY, GPD, JD, GG, SA, KW, VD, KBB, JC, RM, HW, KG.

Methodology–MLY, JC, KBB, GG, JD, GPD, SA, KW, VD, RM, HW, KG.

Supervision–JC, KBB, MLY.

Writing–MLY, GG, JD, GPD, SA, KW, VD, KBB, JC, RM, HW, KG.

Original Draft–MLY, JC, KBB.

Writing–review and editing MLY, JC, KBB, GG, JD, GPD, SA, KW, VD, RM, HW, KG.

GG and JD have accessed and verified the data.

All authors were responsible for the decision to submit the manuscript.

## Data sharing statement

Study protocol and analytic code will be available until 3 years post publication. Following Indigenous data sovereignty best practice and the specifications of our ethics boards and data custodians, individual participant data are not available for sharing. For further information please contact the corresponding author.

## Declaration of interests

MLY is funded by a National Health and Medical Research Council Investigator Grant (APP 2018108). KG is a paid member of the Cancer Australia Research Data Advisory Committee. HW has received consulting fees from Astellas, Boston Scientific and Ferring. HW is also Board directory (unpaid) for the Australian and New Zealand Urogenital and Prostate Cancer Trials Group, Councillor for the Royal Australasian College of Surgeons (unpaid), Baord Director Australasian Urological Foundation (unpaid), Committee chair of the NSW Prostate Outcomes Registry Steering Committee (unpaid) and Editor in Chief, Society of International Urology Journal (paid). All other authors have no conflicts to declare.

All other authors have no conflicts to declare.
