# Supplementary material for: Treatment utilisation for Aboriginal and Torres Strait Islander people diagnosed with prostate cancer in Australia: a population-based study
Source: Lancet Reg Health West Pac. 2026 Aug 4;73:101931. doi: 10.1016/j.lanwpc.2026.101931 (PMC13540680; doi:10.1016/j.lanwpc.2026.101931)

Supplementary Figures

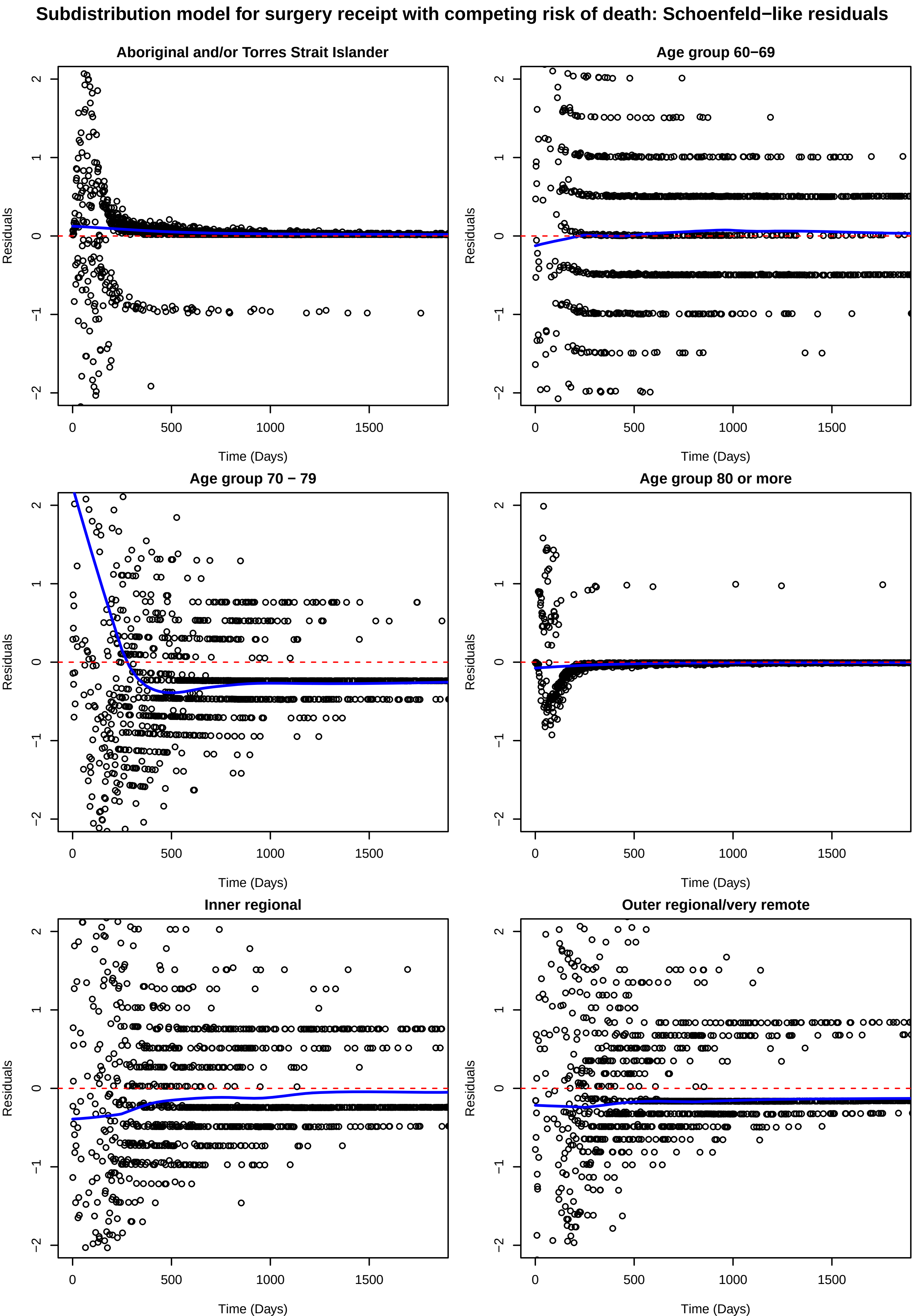

Subdistribution model for surgery receipt with competing risk of death: Schoenfeld−like residuals

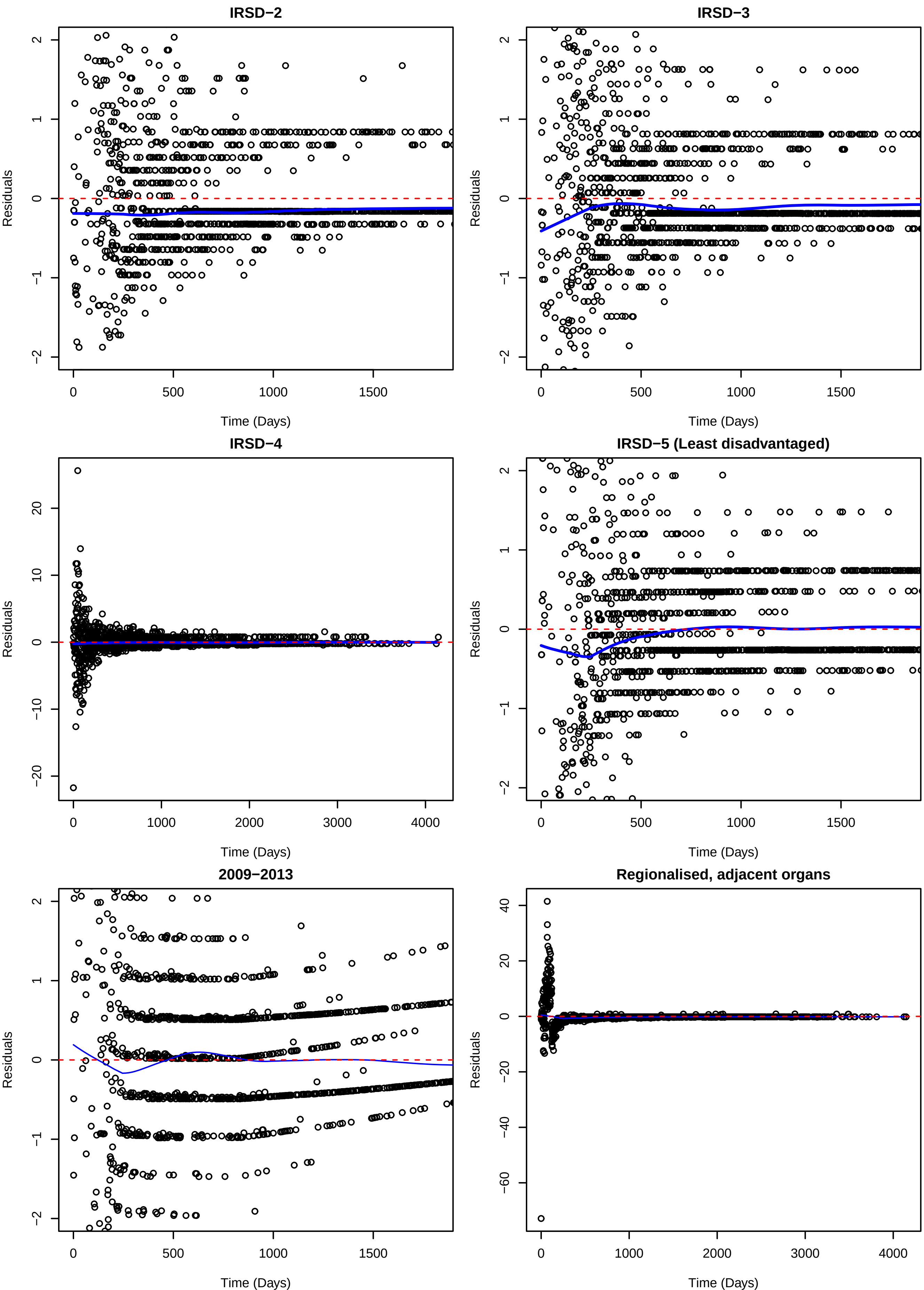

Subdistribution model for surgery receipt with competing risk of death: Schoenfeld−like residuals

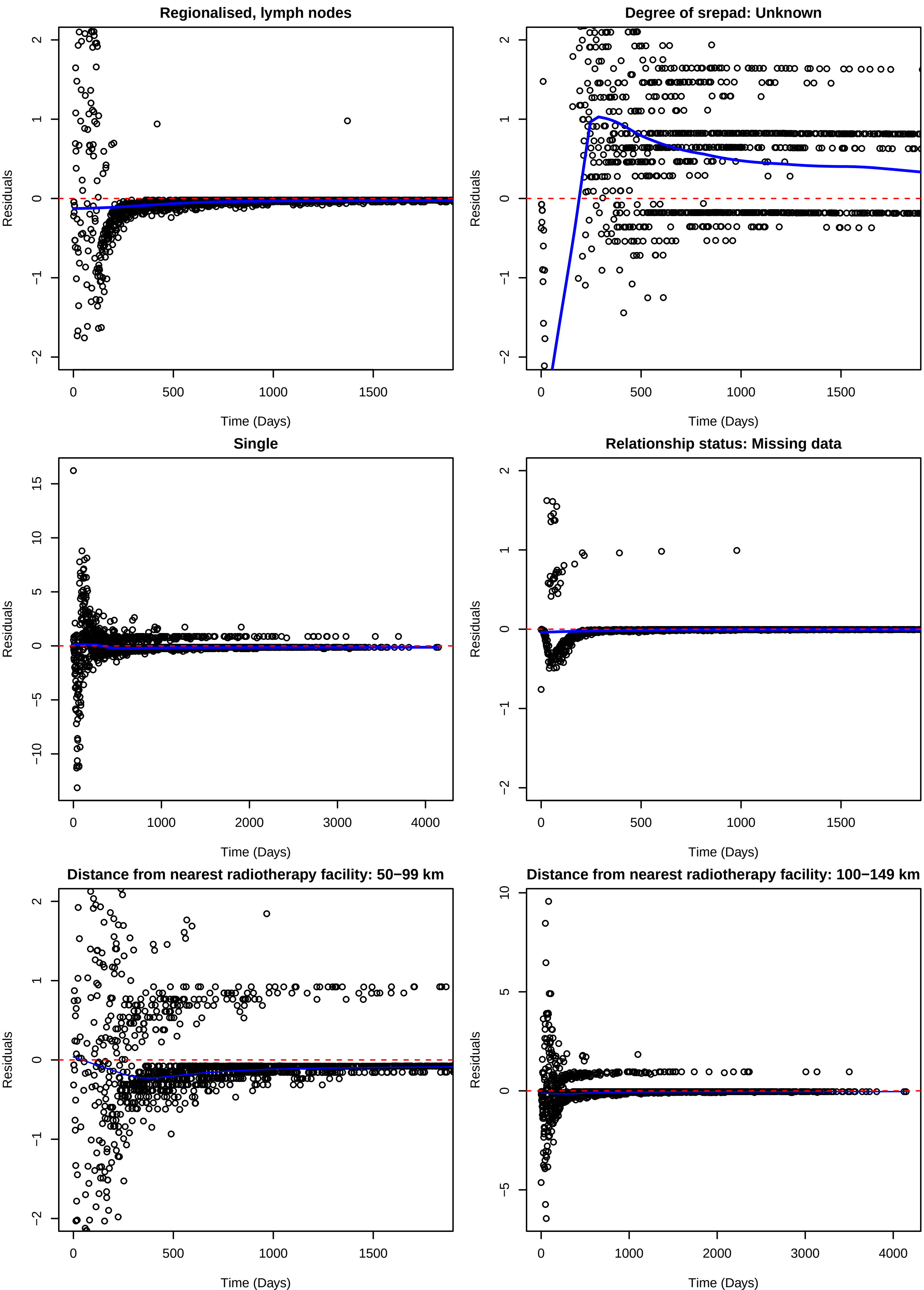

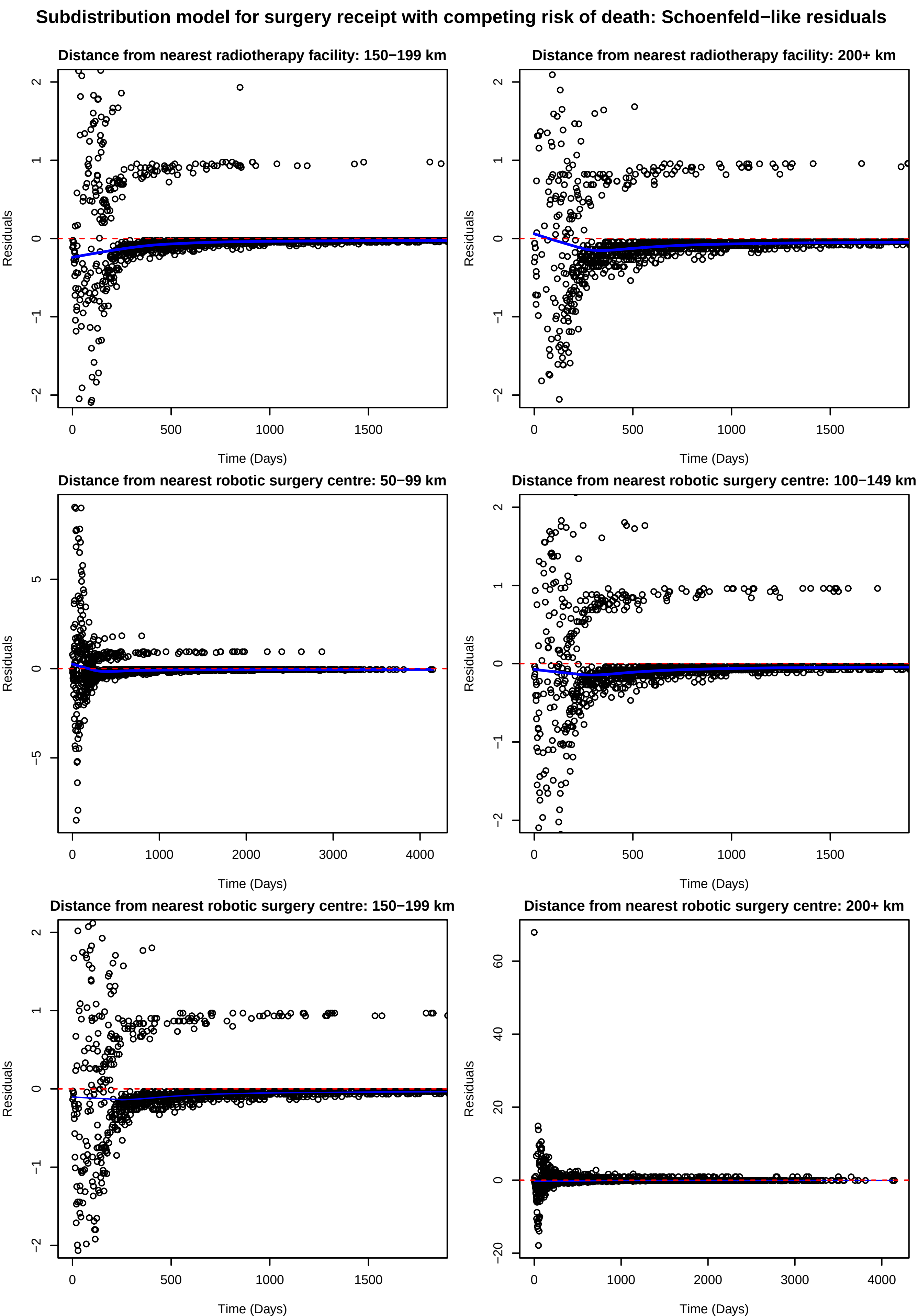
Subdistribution model for surgery receipt with competing risk of death: Schoenfeld−like residuals
Distance from nearest radiotherapy facility: 150−199 km
Distance from nearest radiotherapy facility: 200+ km
Distance from nearest robotic surgery centre: 50−99 km
Distance from nearest robotic surgery centre: 100−149 km
Distance from nearest robotic surgery centre: 150−199 km
Distance from nearest robotic surgery centre: 200+ km
Residuals
Time (Days)

Subdistribution model for surgery receipt with competing risk of death: Schoenfeld−like residuals

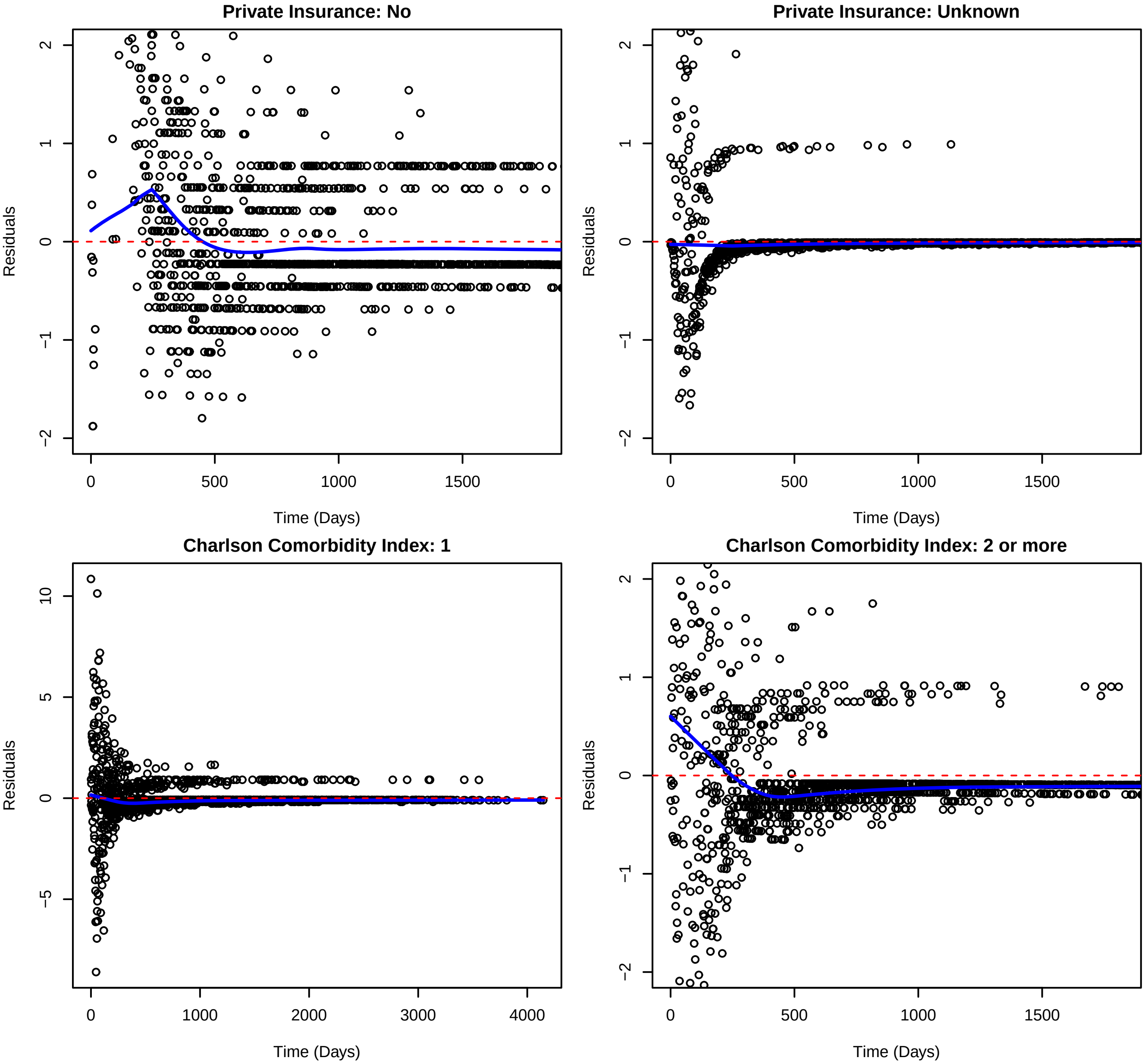

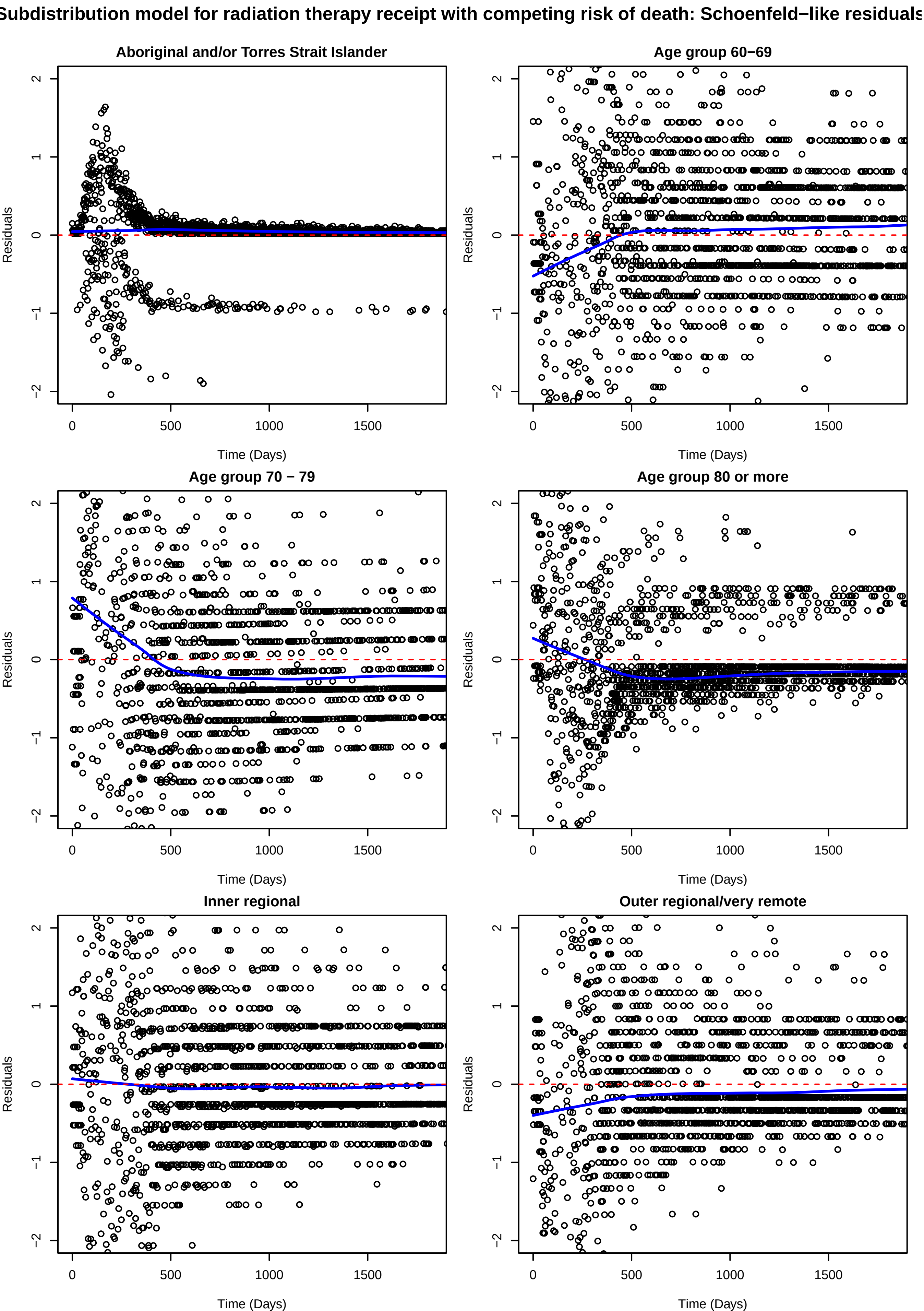
Subdistribution model for radiation therapy receipt with competing risk of death: Schoenfeld−like residuals
Aboriginal and/or Torres Strait Islander
Age group 60−69
Age group 70 − 79
Age group 80 or more
Inner regional
Outer regional/very remote
Residuals
Time (Days)
0
500
1000
1500
−2
−1
0
1
2

Subdistribution model for radiation therapy receipt with competing risk of death: Schoenfeld−like residuals

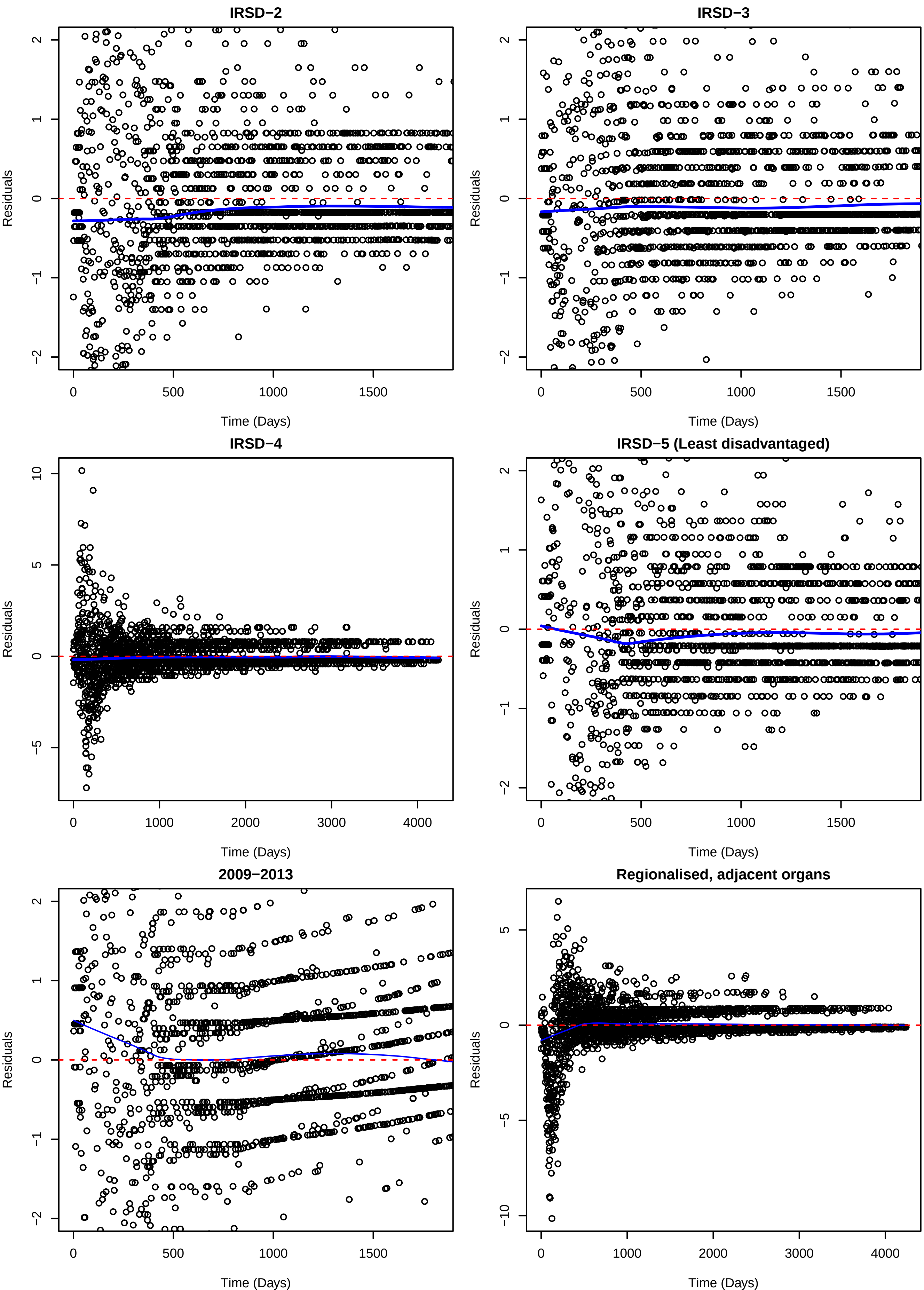

Subdistribution model for radiation therapy receipt with competing risk of death: Schoenfeld−like residuals

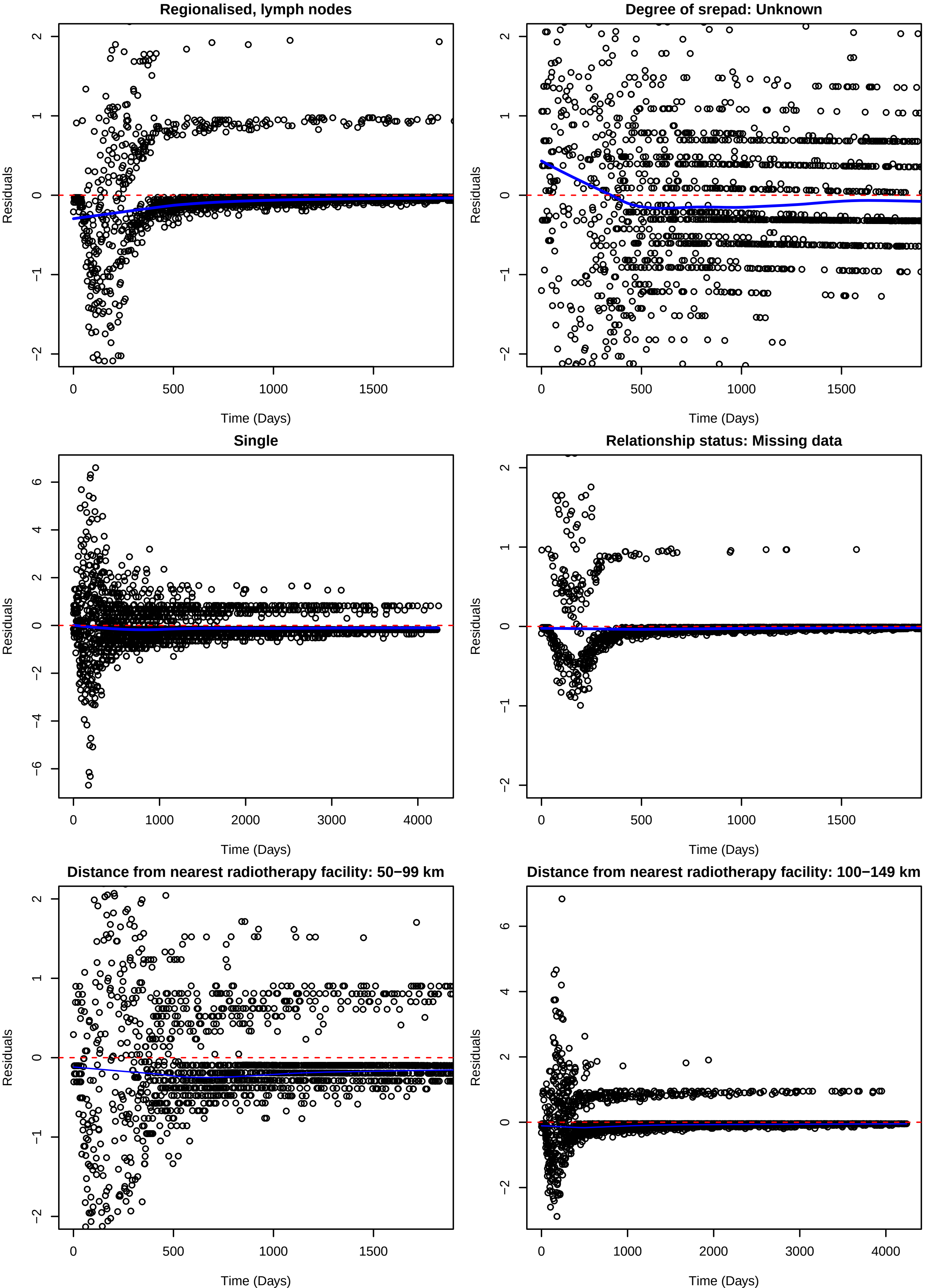

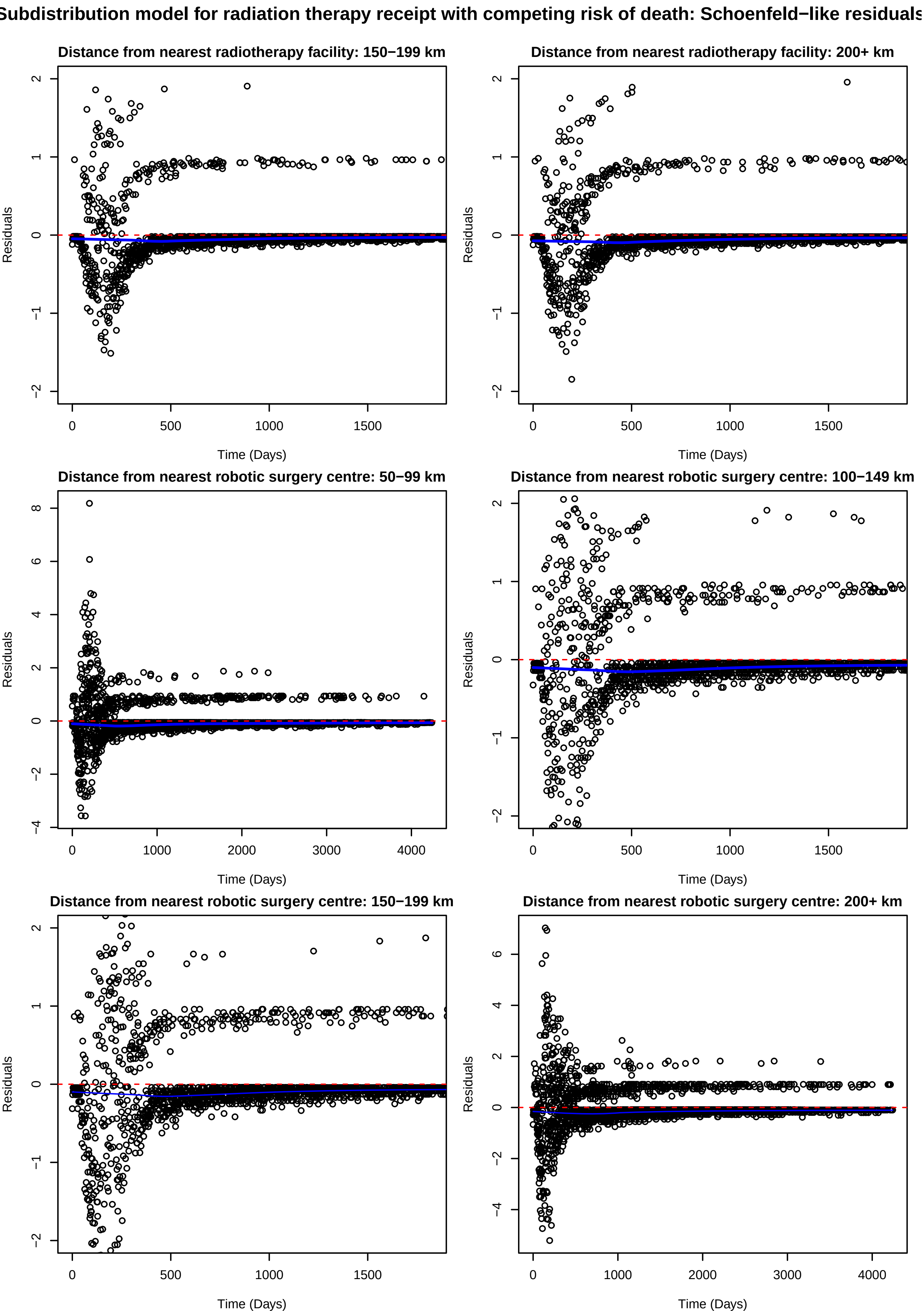

Subdistribution model for radiation therapy receipt with competing risk of death: Schoenfeld−like residuals
Distance from nearest radiotherapy facility: 150−199 km
Distance from nearest radiotherapy facility: 200+ km
Distance from nearest robotic surgery centre: 50−99 km
Distance from nearest robotic surgery centre: 100−149 km
Distance from nearest robotic surgery centre: 150−199 km
Distance from nearest robotic surgery centre: 200+ km
Residuals
Time (Days)

Subdistribution model for radiation therapy receipt with competing risk of death: Schoenfeld−like residuals

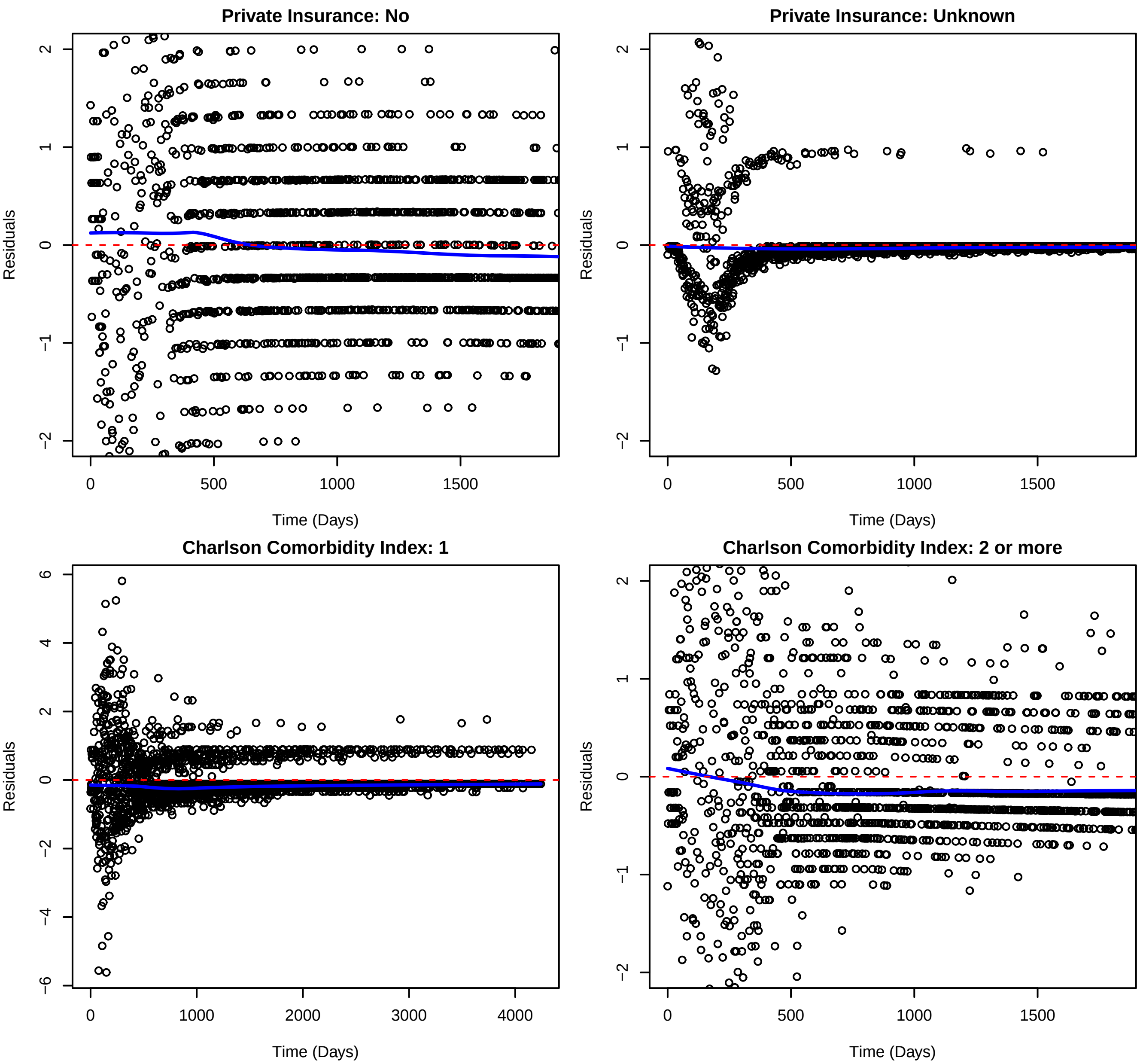

Cox regression analysis on overall survival: Schoenfeld residuals

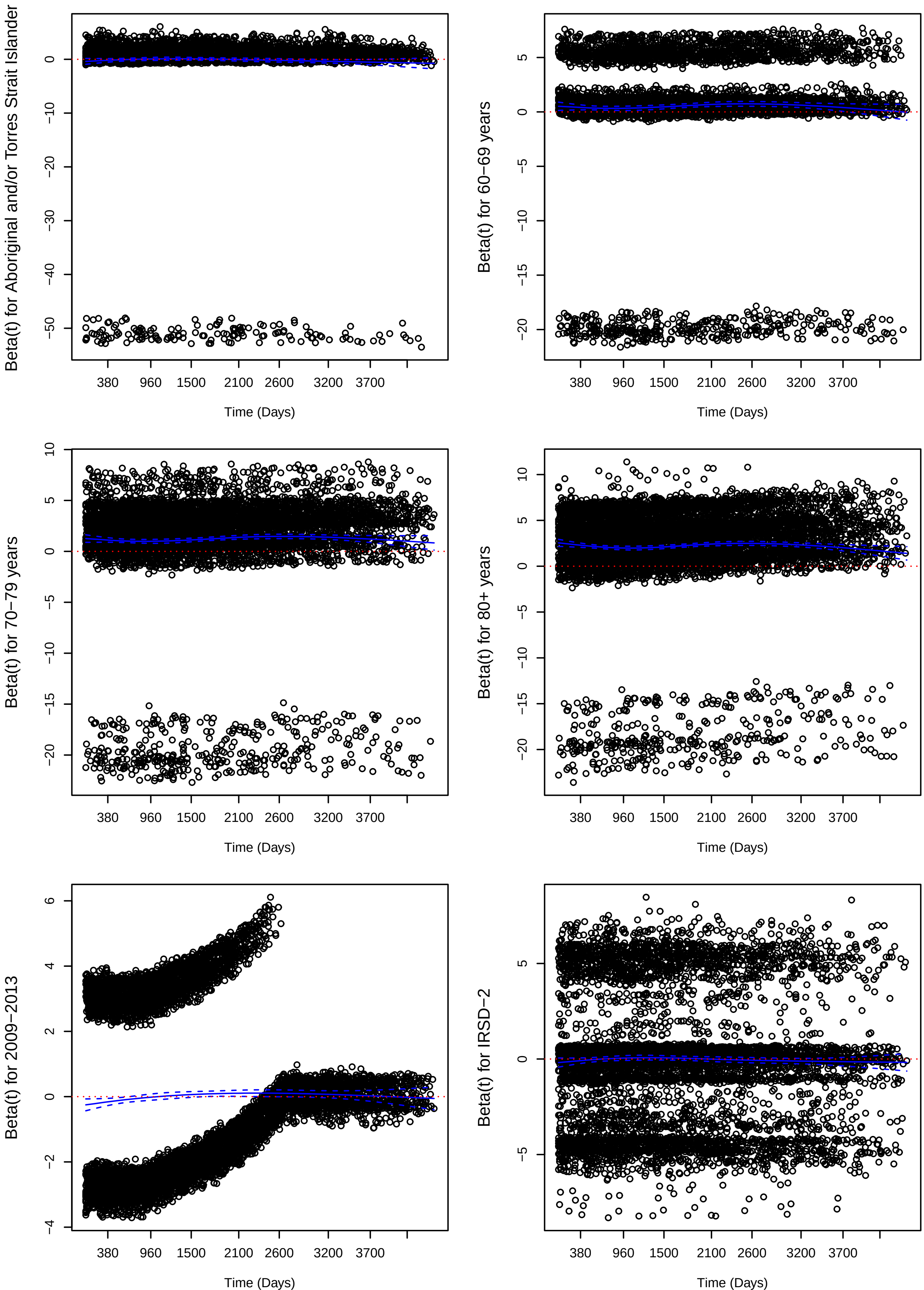

Cox regression analysis on overall survival: Schoenfeld residuals

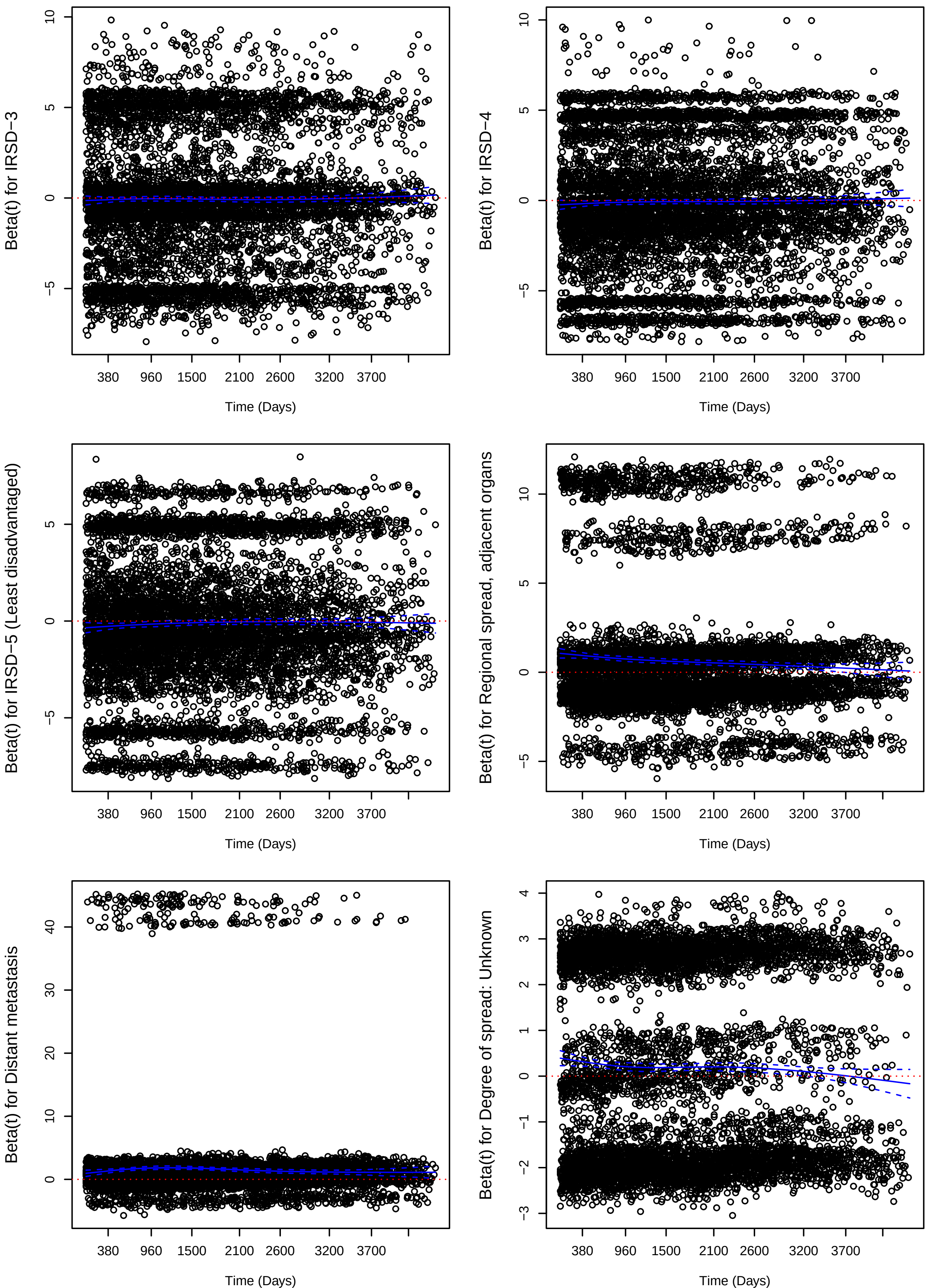

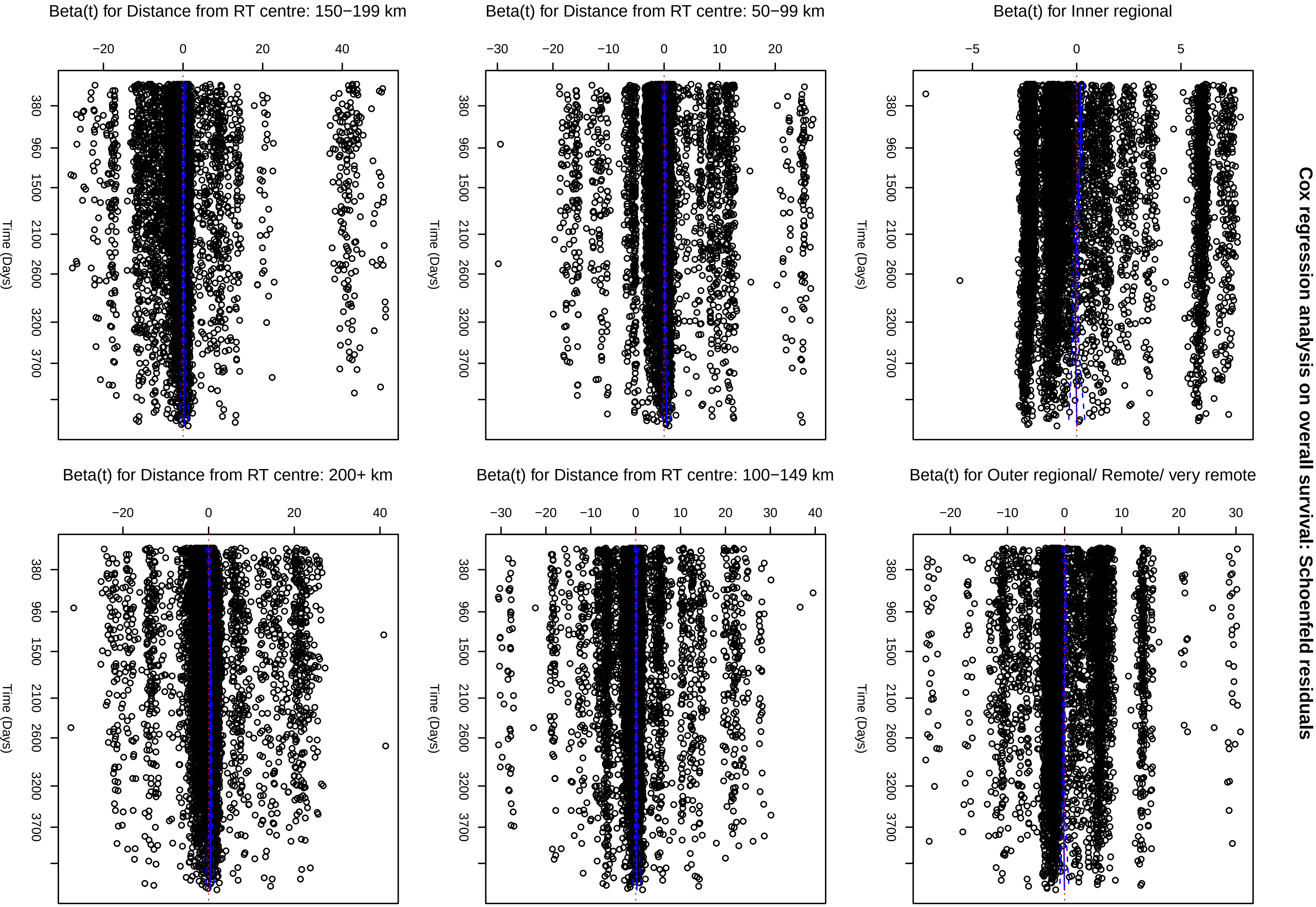
Cox regression analysis on overall survival: Schoenfeld residuals
Beta(t) for Distance from RT centre: 150−199 km
Beta(t) for Distance from RT centre: 50−99 km
Beta(t) for Inner regional
Beta(t) for Distance from RT centre: 200+ km
Beta(t) for Distance from RT centre: 100−149 km
Beta(t) for Outer regional/ Remote/ very remote
Time (Days)
380
960
1500
2100
2600
3200
3700

Cox regression analysis on overall survival: Schoenfeld residuals

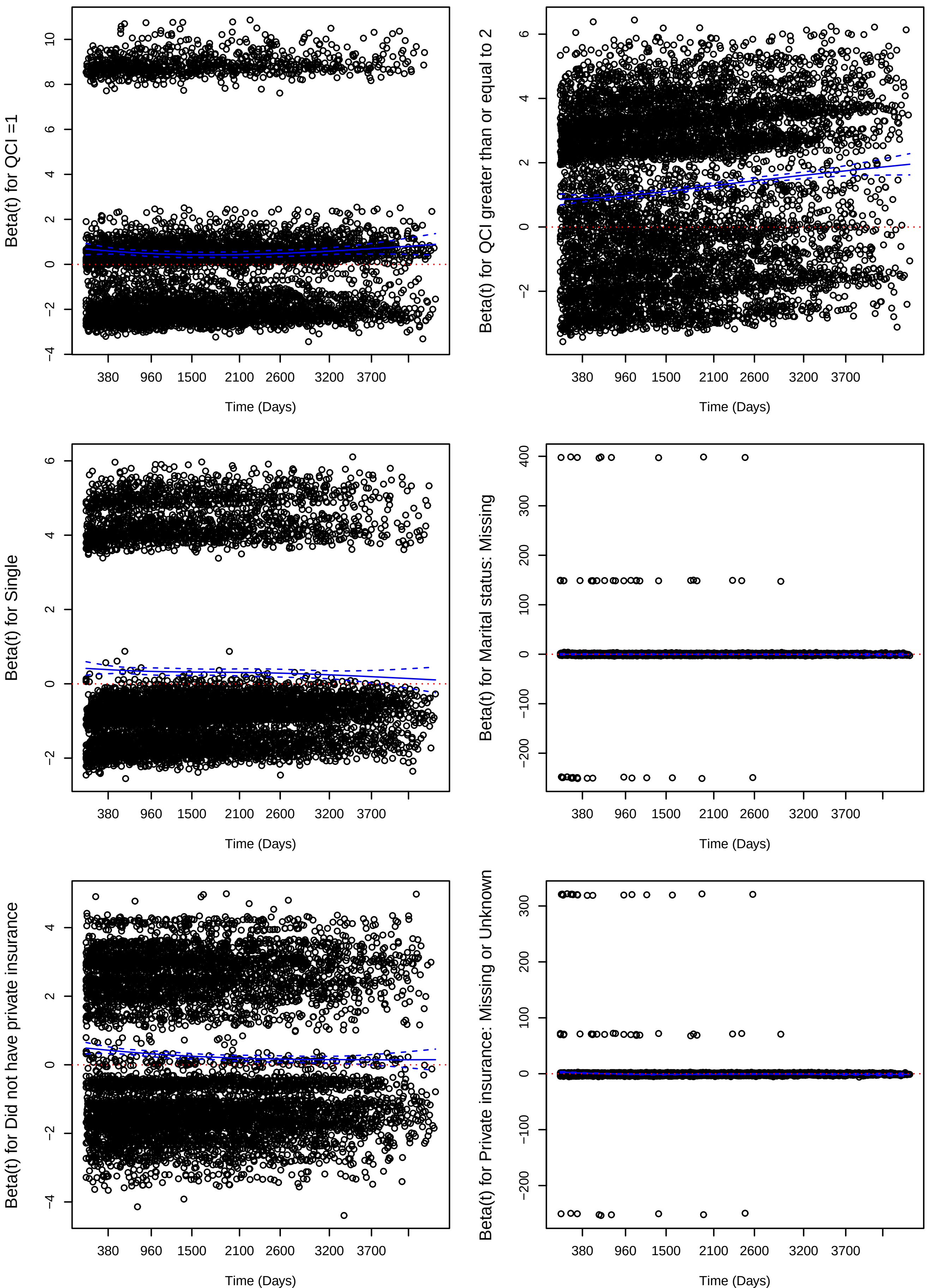

Cox regression analysis on overall survival: Schoenfeld residuals

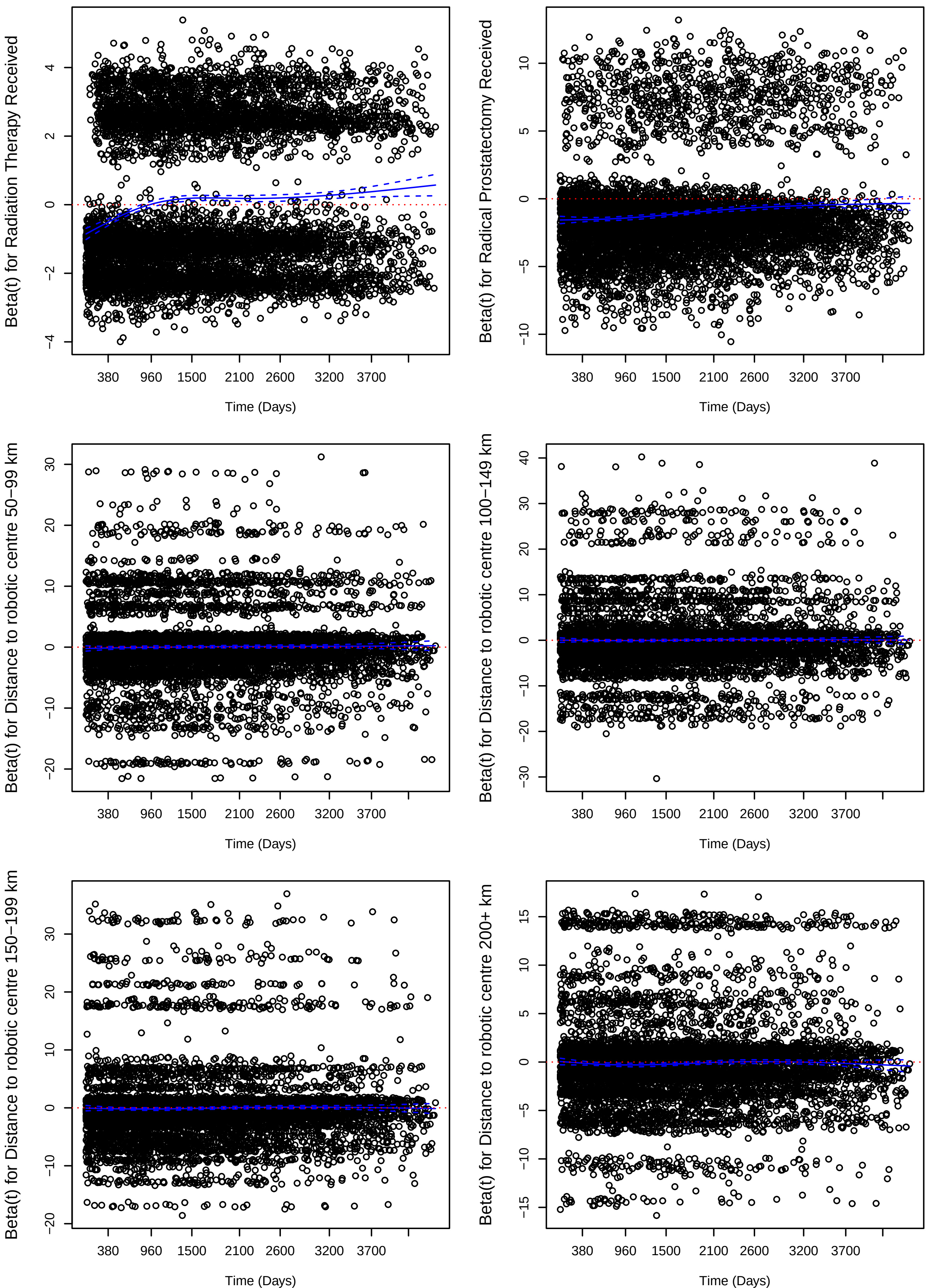

Cox regression analysis on cancer specific mortality: Schoenfeld residuals

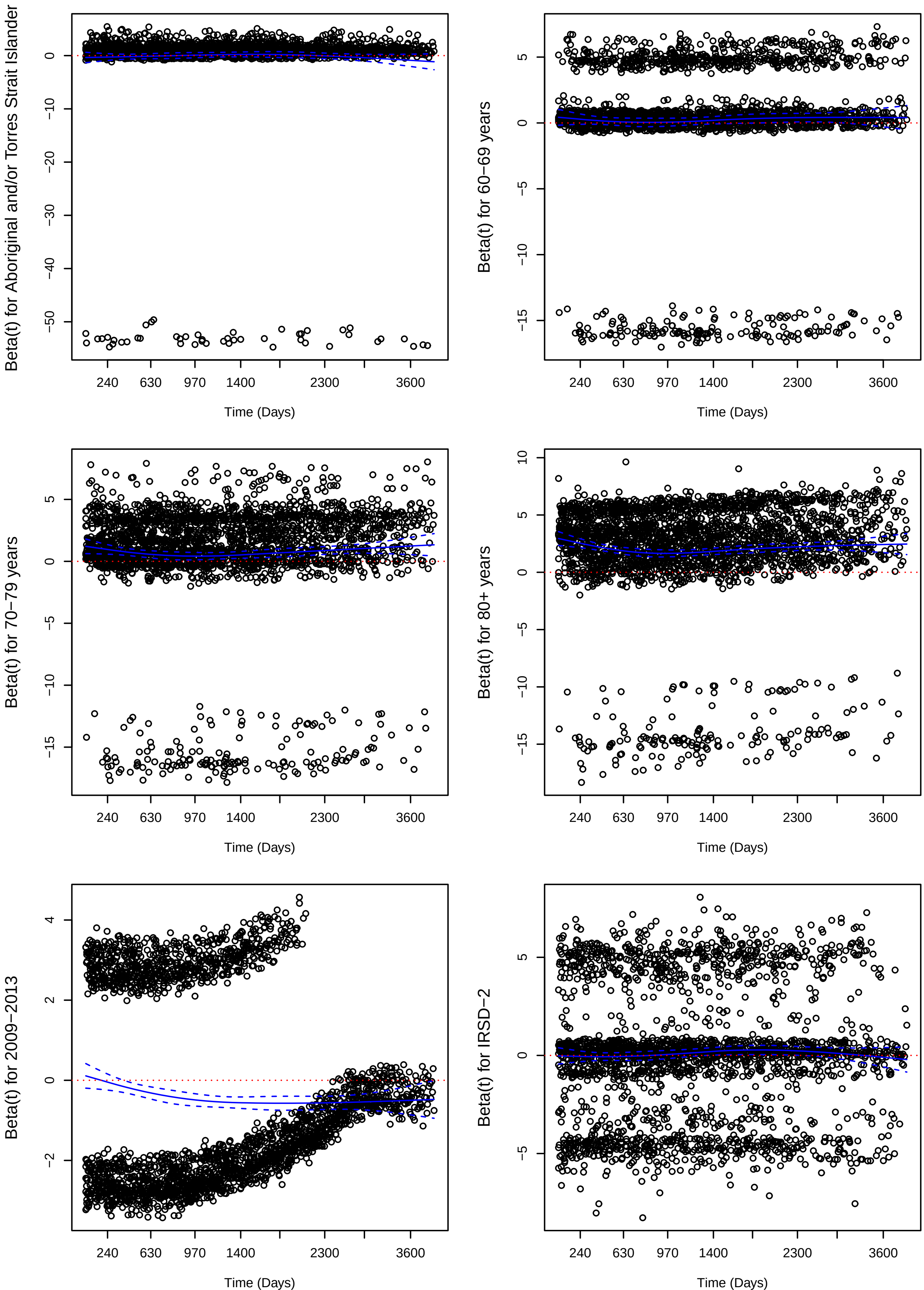

Cox regression analysis on cancer specific mortality: Schoenfeld residuals

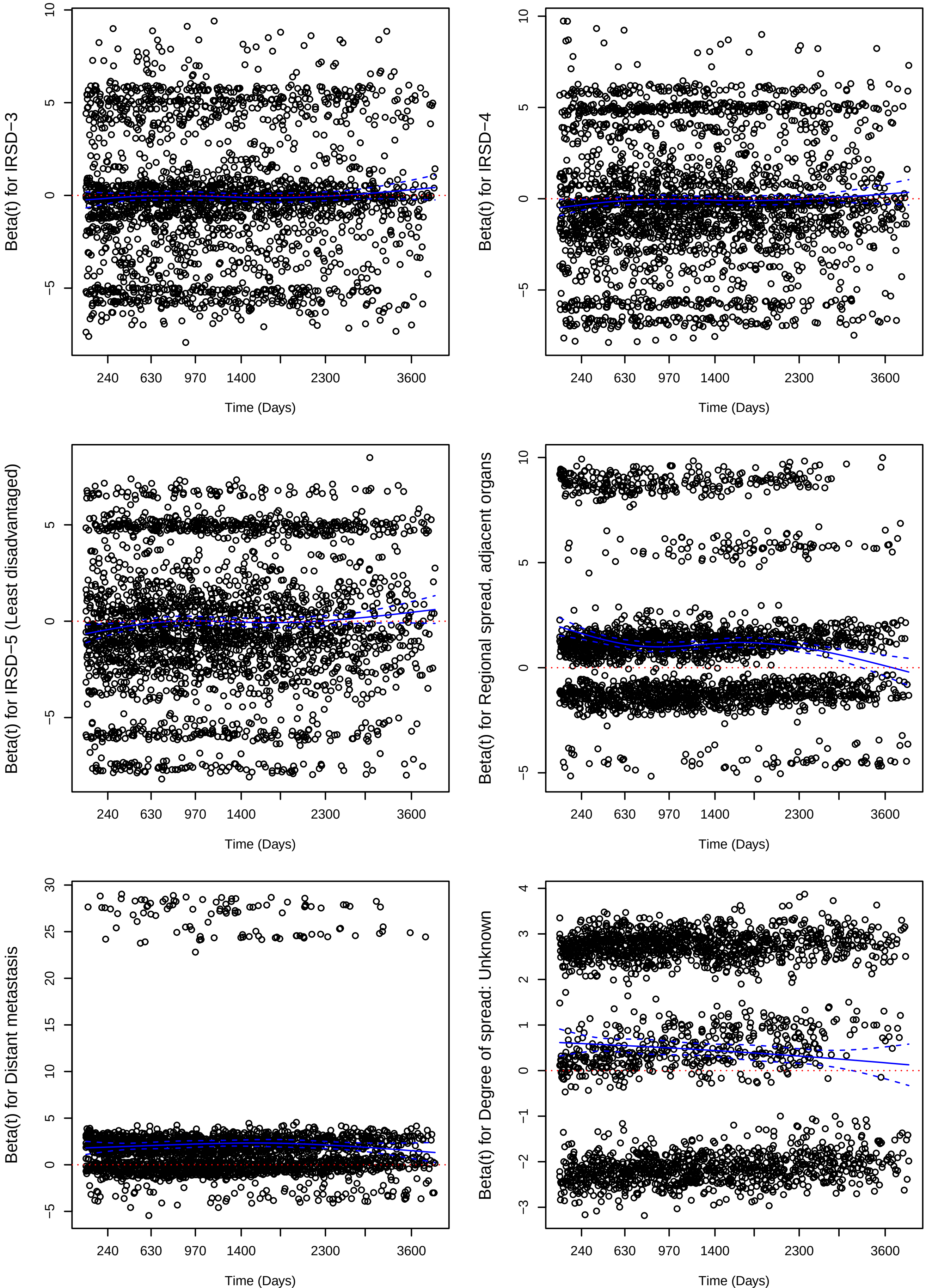

Cox regression analysis on cancer specific mortality: Schoenfeld residuals

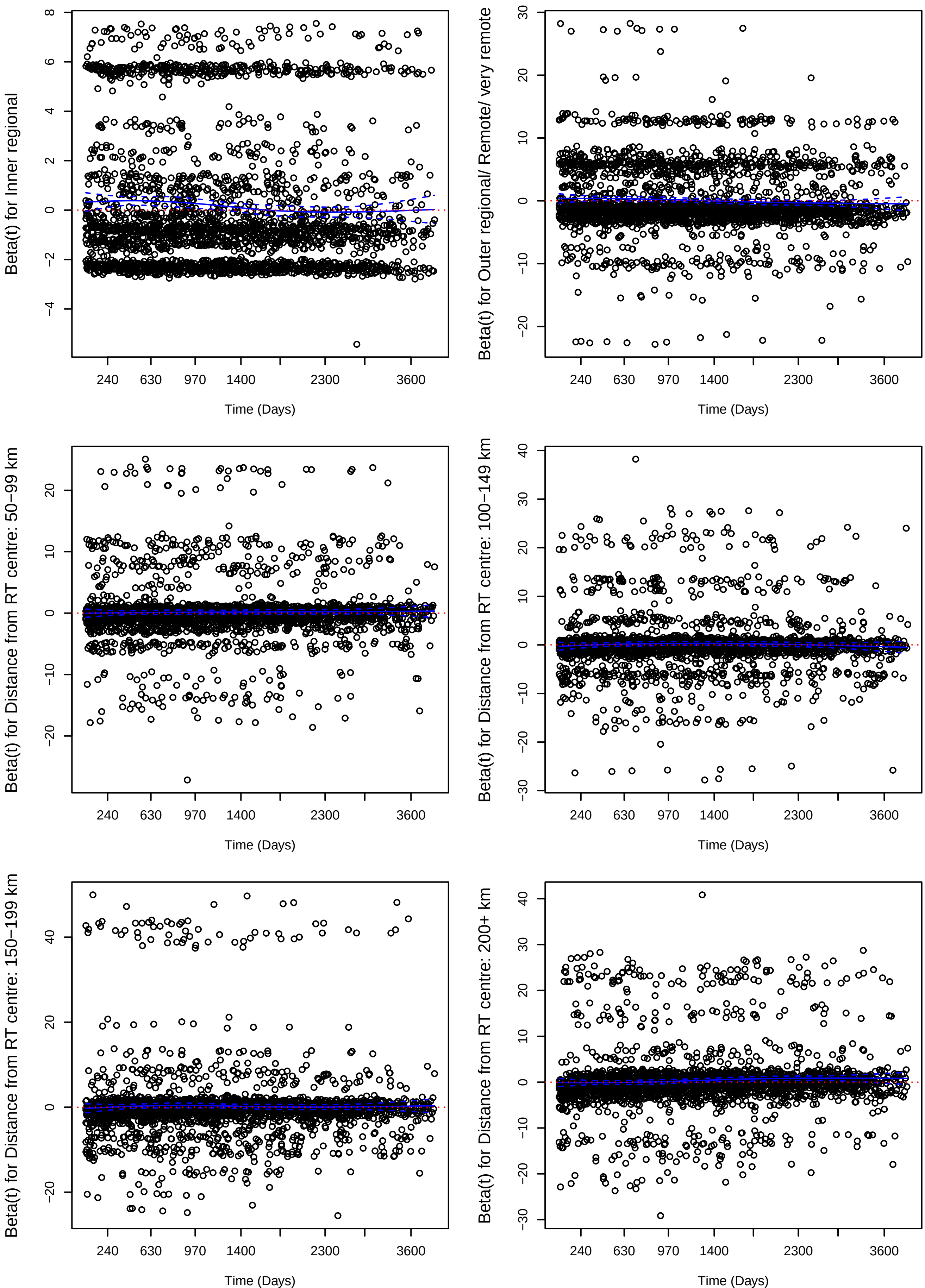

Cox regression analysis on cancer specific mortality: Schoenfeld residuals

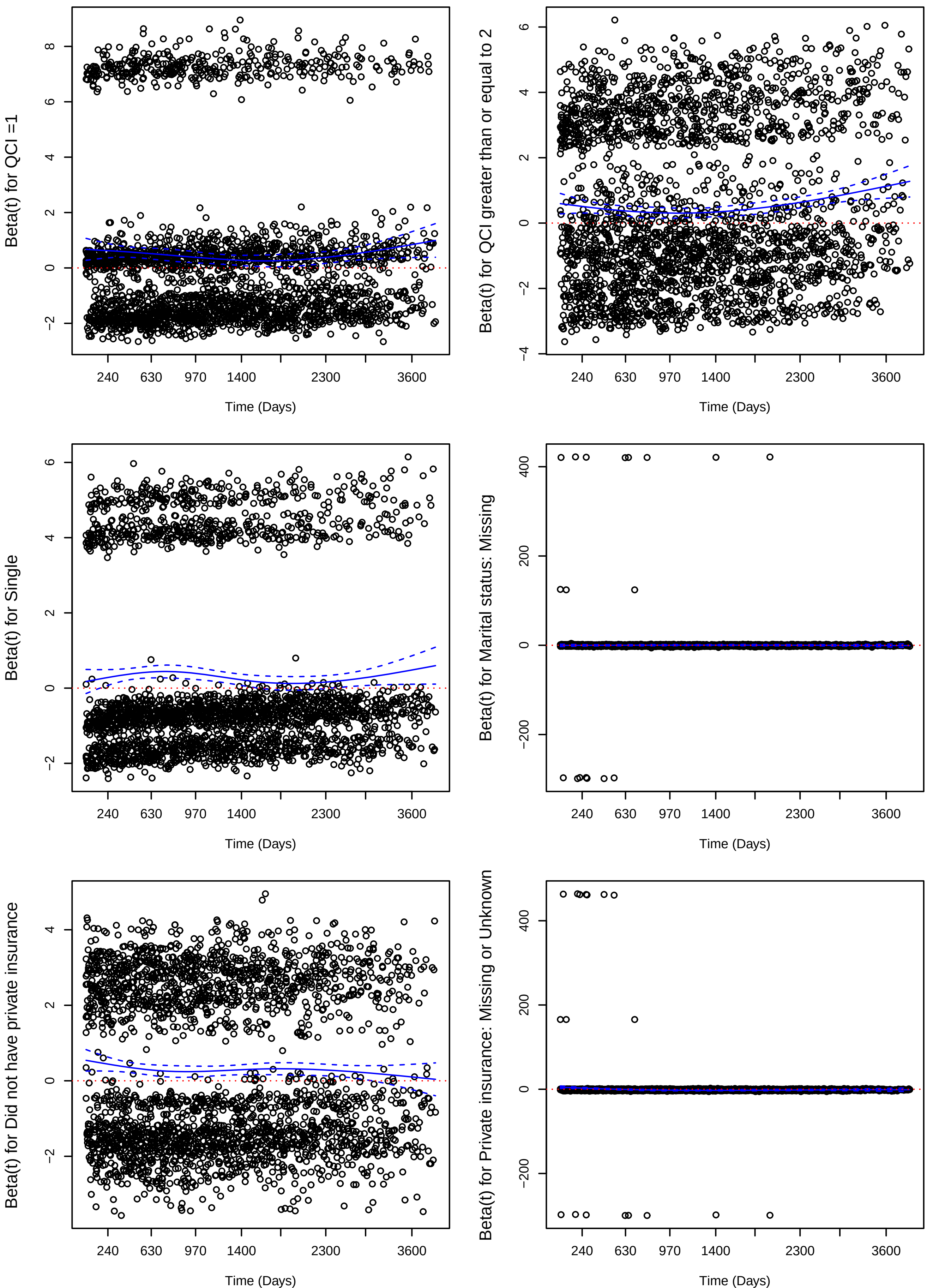

Cox regression analysis on cancer specific mortality: Schoenfeld residuals

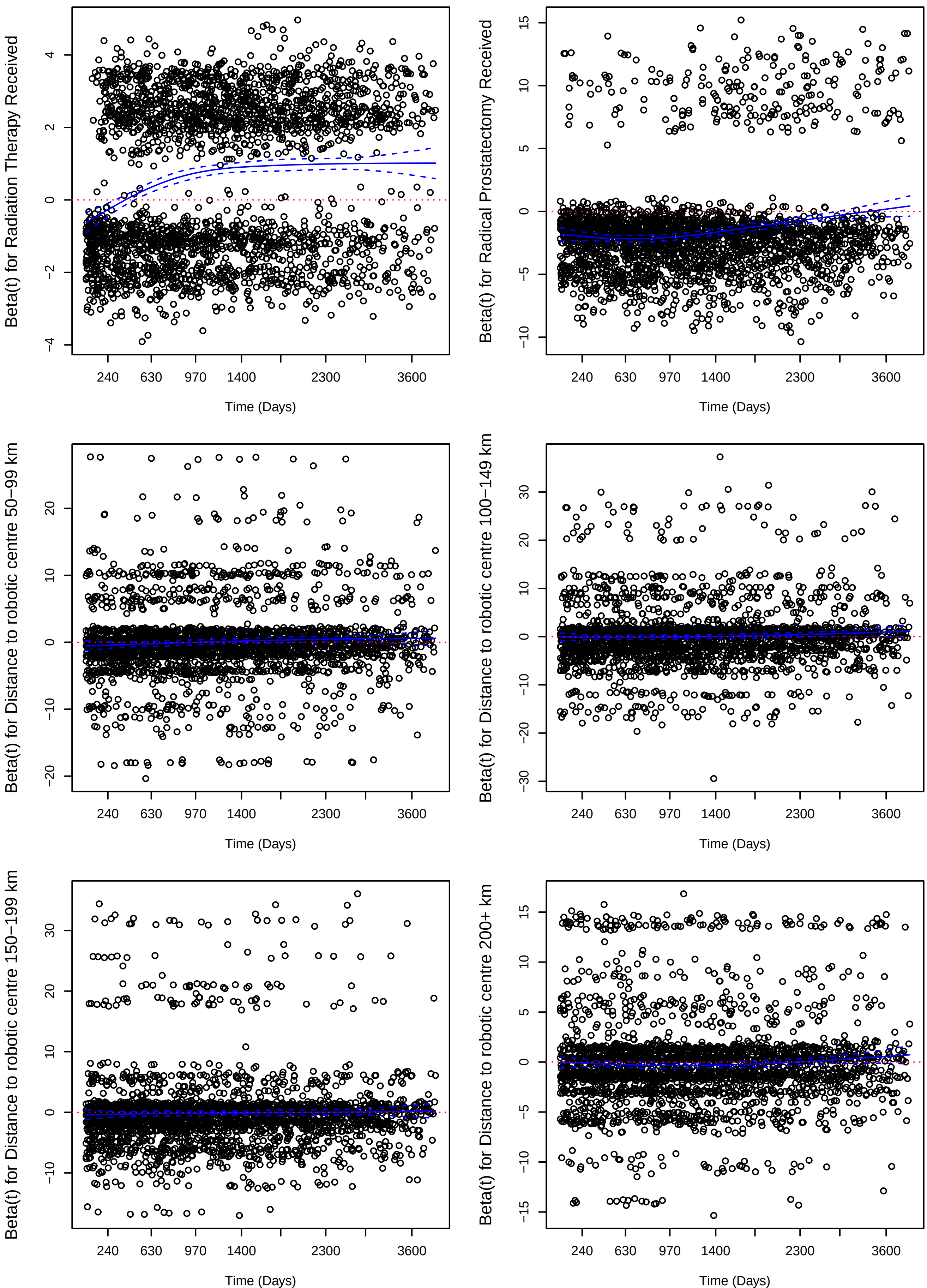

Supplement: Supplementary file 1 [file mmc2.pdf]
