# Supplementary material for: Treatment utilisation for Aboriginal and Torres Strait Islander people diagnosed with prostate cancer in Australia: a population-based study
Source: Lancet Reg Health West Pac. 2026 Aug 4;73:101931. doi: 10.1016/j.lanwpc.2026.101931 (PMC13540680; doi:10.1016/j.lanwpc.2026.101931)
Supplement: Supplementary file 2 [file mmc1.docx]

**Supplementary tables**

**Supplementary table 1 – ICD-10AM procedure codes for prostate cancer treatment**

| **Procedure** | |
| --- | --- |
| Codes |  |
| 37209-00, 37210-00, 37211-00, 37209-01, 37210-01, 37211-01 | Prostatectomy |
| 15000-00, 15003-00,15100-00, 15103-00, 15224-00, 15239-00, 15254-00, 15269-00, 15600-00,15600-01,15600-02,15600-03,15600-04, 15600-00,15600-01,15600-02,15600-03,15600-04 | External beam radiotherapy |
| 15338-00, 15539-00, 37227-00. 90764-00, 90764-01 | Brachytherapy |

Supplementary table 2a – Period Specific Analysis, Subdistribution model for surgery receipt with competing risk of death

|  | 0-12 months | | | 12+ months | | |
| --- | --- | --- | --- | --- | --- | --- |
| **Sociodemographic or health factor** | HR | 95% CI | p-value | HR | 95% CI | p-value |
| **Aboriginal and Torres Strait Islander identification** |  |  |  |  |  |  |
| Aboriginal and/or Torres Strait Islander | 0.92 | (0.82, 1.03) | 0.134 | 0.94 | (0.69, 1.28) | 0.688 |
| Non-Indigenous | Reference |  |  | Reference |  |  |
| **Age at diagnosis (years)** |  |  | <0.001 |  |  | <0.001 |
| <60 | Reference |  |  | Reference |  |  |
| 60-69 | 0.84 | (0.81, 0.86) |  | 0.66 | (0.61, 0.73) |  |
| 70-79 | 0.4 | (0.38, 0.42) |  | 0.15 | (0.13, 0.18) |  |
| 80 or more | 0.025 | (0.02, 0.03) |  | 0.008 | (0.00, 0.02) |  |
| **Place of residence** |  |  | 0.048 |  |  | 0.508 |
| Major cities | Reference |  |  | Reference |  |  |
| Inner regional | 1.05 | (1.01, 1.09) |  | 0.98 | (0.87, 1.1) |  |
| Outer regional/remote/very remote | 1.02 | (0.93, 1.11) |  | 0.86 | (0.67, 1.11) |  |
| **Index of Relative Socioeconomic Disadvantage** |  |  | <0.001 |  |  | <0.001 |
| IRSD-1 (Most disadvantaged) | Reference |  |  | Reference |  |  |
| IRSD-2 | 1.06 | (1.00, 1.11) |  | 1.12 | (0.96, 1.32) |  |
| IRSD-3 | 1.11 | (1.05, 1.17) |  | 1.16 | (0.99, 1.37) |  |
| IRSD-4 | 1.18 | (1.12, 1.24) |  | 1.33 | (1.13, 1.56) |  |
| IRSD-5 (Least disadvantaged) | 1.34 | (1.27, 1.41) |  | 1.45 | (1.24, 1.71) |  |
| **Year of Diagnosis** |  |  | <0.001 |  |  | 0.098 |
| 2009-2013 | 1.06 | (1.03, 1.09) |  | 1.08 | (0.99, 1.18) |  |
| 2014-2018 | Reference |  |  | Reference |  |  |
| **Degree of spread** |  |  | <0.001 |  |  | <0.001 |
| Localised | Reference |  |  | Reference |  |  |
| Regionalised, adjacent organs | 3.39 | (3.28, 3.51) |  | 0.3 | (0.20, 0.45) |  |
| Regionalised, lymph nodes | 3.41 | (3.11, 3.73) |  | 0.28 | (0.09, 0.85) |  |
| Unknown | 0.3 | (0.29, 0.31) |  | 1.23 | (1.13, 1.34) |  |
| **Relationship status** |  |  | <0.001 |  |  | <0.001 |
| Married/partnered | Reference |  |  | Reference |  |  |
| Single | 0.75 | (0.72, 0.78) |  | 0.69 | (0.60, 0.79) |  |
| Missing data | 0.22 | (0.16, 0.31) |  | 0.091 | (0.02, 0.34) |  |
| **Distance from nearest radiotherapy facility** |  |  | <0.001 |  |  | <0.001 |
| <50 km | Reference |  |  | Reference |  |  |
| 50-99 km | 1.06 | (0.97, 1.16) |  | 1.36 | (1.06, 1.75) |  |
| 100-149 km | 1.36 | (1.22, 1.51) |  | 1.67 | (1.25, 2.22) |  |
| 150-199 km | 1.49 | (1.31, 1.69) |  | 1.84 | (1.30, 2.59) |  |
| 200+ km | 2.37 | (2.12, 2.64) |  | 2.21 | (1.61, 3.03) |  |
| **Distance from nearest robotic surgery centre** |  |  | <0.001 |  |  | 0.005 |
| <50 km | Reference |  |  | Reference |  |  |
| 50-99 km | 0.82 | (0.75, 0.91) |  | 0.55 | (0.40, 0.75) |  |
| 100-149 km | 0.72 | (0.64, 0.80) |  | 0.86 | (0.65, 1.16) |  |
| 150-199 km | 0.76 | (0.69, 0.84) |  | 0.85 | (0.64, 1.12) |  |
| 200+ km | 0.68 | (0.63, 0.74) |  | 0.94 | (0.77, 1.14) |  |
| **Health insurance status** |  |  | <0.001 |  |  | <0.001 |
| Private health insurance | Reference |  |  | Reference |  |  |
| No private insurance | 0.65 | (0.63, 0.68) |  | 0.62 | (0.56, 0.70) |  |
| Unknown/missing | 0.61 | (0.51, 0.74) |  | 0.49 | (0.26, 0.91) |  |
| **Charlson comorbidity index (Quan)** |  |  | <0.001 |  |  | <0.001 |
| 0 | Reference |  |  | Reference |  |  |
| 1 | 0.81 | (0.77, 0.86) |  | 0.72 | (0.61, 0.84) |  |
| 2 or more | 0.57 | (0.54, 0.61) |  | 0.41 | (0.34, 0.50) |  |

Supplementary table 2b – Period Specific Analysis, Subdistribution model for radiotherapy receipt with competing risk of death

|  | 0-12 months | | | 12+ months | | |
| --- | --- | --- | --- | --- | --- | --- |
| **Sociodemographic or health factor** | HR | 95% CI | p-value | HR | 95% CI | p-value |
| **Aboriginal and Torres Strait Islander identification** |  |  |  |  |  |  |
| Aboriginal and/or Torres Strait Islander | 1.24 | (1.09, 1.42) | 0.001 | 1.20 | (0.98, 1.47) | 0.086 |
| Non-Indigenous | Reference |  |  | Reference |  |  |
| **Age at diagnosis (years)** |  |  | <0.001 |  |  | <0.001 |
| <60 | Reference |  |  | Reference |  |  |
| 60-69 | 1.87 | (1.75, 2.00) |  | 1.40 | (1.30, 1.52) |  |
| 70-79 | 3.63 | (3.39, 3.88) |  | 1.69 | (1.55, 1.84) |  |
| 80 or more | 1.9 | (1.73, 2.09) |  | 1.05 | (0.92, 1.11) |  |
| **Place of residence** |  |  | <0.001 |  |  | <0.001 |
| Major cities | Reference |  |  | Reference |  |  |
| Inner regional | 1.09 | (1.04, 1.15) |  | 1.11 | (1.02, 1.19) |  |
| Outer regional/remote/very remote | 1.42 | (1.28, 1.58) |  | 1.44 | (1.23, 1.69) |  |
| **Index of Relative Socioeconomic Disadvantage** |  |  | <0.001 |  |  | <0.001 |
| IRSD-1 (Most disadvantaged) | Reference |  |  | Reference |  |  |
| IRSD-2 | 0.92 | (0.86, 0.98) |  | 1.00 | (0.91, 1.10) |  |
| IRSD-3 | 0.97 | (0.91, 1.03) |  | 1.02 | (0.93, 1.12) |  |
| IRSD-4 | 0.83 | (0.78, 0.88) |  | 0.99 | (0.90, 1.09) |  |
| IRSD-5 (Least disadvantaged) | 0.75 | (0.70, 0.80) |  | 0.86 | (0.78, 0.94) |  |
| **Year of Diagnosis** |  |  |  |  |  |  |
| 2009-2013 | 0.86 | (0.83, 0.90) | <0.001 | 0.85 | (0.81, 0.91) | <0.001 |
| 2014-2018 | Reference |  |  | Reference |  |  |
| **Degree of spread** |  |  | <0.001 |  |  | <0.001 |
| Localised | Reference |  |  | Reference |  |  |
| Regionalised, adjacent organs | 1.11 | (1.05, 1.17) |  | 2.14 | (2.00, 2.30) |  |
| Regionalised, lymph nodes | 1.74 | (1.53, 1.97) |  | 3.53 | (3.02, 4.11) |  |
| Unknown | 1.14 | (1.09, 1.19) |  | 1.22 | (1.13, 1.30) |  |
| **Relationship status** |  |  | <0.001 |  |  | 0.609 |
| Married/partnered | Reference |  |  | Reference |  |  |
| Single | 1.01 | (0.96, 1.07) |  | 1.04 | (0.96, 1.12) |  |
| Missing data | 1.83 | (1.51, 2.21) |  | 0.93 | (0.62, 1.39) |  |
| **Distance from nearest treatment facility** |  |  | <0.001 |  |  | <0.001 |
| <50 km | Reference |  |  | Reference |  |  |
| 50-99 km | 0.77 | (0.69, 0.85) |  | 0.74 | (0.64, 0.86) |  |
| 100-149 km | 0.62 | (0.55, 0.70) |  | 0.66 | (0.56, 0.79) |  |
| 150-199 km | 0.44 | (0.37, 0.52) |  | 0.52 | (0.41, 0.66) |  |
| 200+ km | 0.21 | (0.18, 0.25) |  | 0.35 | (0.28, 0.43) |  |
| **Distance from nearest robotic surgery centre** |  |  | 0.016 |  |  | 0.273 |
| <50 km | Reference |  |  | Reference |  |  |
| 50-99 km | 1.16 | (1.03, 1.30) |  | 1.12 | (0.95, 1.33) |  |
| 100-149 km | 1.05 | (0.93, 1.20) |  | 1.10 | (0.91, 1.32) |  |
| 150-199 km | 1.01 | (0.90, 1.13) |  | 1.14 | (0.96, 1.36) |  |
| 200+ km | 0.95 | (0.88, 1.04) |  | 1.15 | (1.01, 1.30) |  |
| **Health insurance status** |  |  | <0.001 |  |  | <0.001 |
| Private health insurance | Reference |  |  | Reference |  |  |
| No private insurance | 1.45 | (1.39, 1.51) |  | 1.27 | (1.19, 1.36) |  |
| Unknown/missing | 1.04 | (0.87, 1.23) |  | 0.71 | (0.51, 1.00) |  |
| **Charlson comorbidity index (Quan)** |  |  | <0.001 |  |  | 0.007 |
| 0 | Reference |  |  | Reference |  |  |
| 1 | 1.13 | (1.06, 1.20) |  | 1.16 | (1.06, 1.27) |  |
| 2 or more | 1.01 | (0.95, 1.06) |  | 0.90 | (0.80, 1.00) |  |

**Supplementary model 3 – Cox regression on overall mortality, time varying analysis**

|  | 0-1 year | | | 1-5 years | | | 5+ years | | |
| --- | --- | --- | --- | --- | --- | --- | --- | --- | --- |
| **Sociodemographic or health factor** | HR | 95% CI | p-value | HR | 95% CI | p-value | HR | 95% CI | p-value |
| **Aboriginal and Torres Strait Islander Identification** |  |  | 0.365 |  |  | 0.577 |  |  | 0.0206 |
| Aboriginal and Torres Strait Islander | 1.18 | (0.82, 1.69) |  | 0.94 | (0.75, 1.18) | | 1.33 | (1.04, 1.69) | |
| Non-Indigenous | Reference |  |  | Reference |  |  | Reference |  |  |
| **Age at diagnosis (years)** |  |  | <0.001 |  |  | <0.001 |  |  | <0.001 |
| <60 | Reference |  |  | Reference |  |  | Reference |  |  |
| 60-69 | 1.33 | (0.99, 1.78) |  | 1.35 | (1.17, 1.56) | | 1.74 | (1.48, 2.05) | |
| 70-79 | 2.31 | (1.75, 3.06) |  | 2.49 | (2.17, 2.85) | | 3.47 | (2.95, 4.08) | |
| 80 or more | 6.07 | (4.61, 7.99) |  | 6.36 | (5.53, 7.31) | | 9.04 | (7.61, 10.74) | |
| **Place of residence** |  |  | 0.038 |  |  | 0.003 |  |  | 0.323 |
| Major cities | Reference |  |  | Reference |  |  | Reference |  |  |
| Inner regional | 1.09 | (0.95, 1.25) |  | 1.16 | (1.07, 1.26) | | 1.00 | (0.91, 1.11) | |
| Outer regional/remote/very remote | 1.21 | (0.93, 1.59) |  | 1.14 | (0.97, 1.34) | | 0.88 | (0.72, 1.07) | |
| **Index of Relative Socioeconomic Disadvantage** |  |  | 0.289 |  |  | 0.252 |  |  | 0.8 |
| IRSD-1 (Most disadvantaged) | Reference |  |  | Reference |  |  | Reference |  |  |
| IRSD-2 | 1.12 | (0.97, 1.31) |  | 1.04 | (0.95, 1.14) | | 0.93 | (0.83, 1.04) | |
| IRSD-3 | 1.00 | (0.85, 1.18) |  | 0.97 | (0.89, 1.07) | | 0.96 | (0.85, 1.08) | |
| IRSD-4 | 1.08 | (0.92, 1.27) |  | 0.98 | (0.89, 1.08) | | 0.98 | (0.87, 1.10) |  |
| IRSD-5 (Least disadvantaged) | 0.96 | (0.81, 1.14) |  | 0.92 | (0.83, 1.02) | | 0.97 | (0.86, 1.09) | |
| **Year of Diagnosis** |  |  | 0.038 |  |  | 0.073 |  |  | 0.09 |
| 2009-2013 | 1.11 | (1.01, 1.22) |  | 1.06 | (1, 1.12) |  | 0.89 | (0.79, 1.02) | |
| 2014-2018 | Reference |  |  | Reference |  |  | Reference |  |  |
| **Degree of spread** |  |  | <0.001 |  |  | <0.001 |  |  | <0.001 |
| Localised | Reference |  |  | Reference |  |  | Reference |  |  |
| Regionalised, adjacent organs | 2.65 | (2.1, 3.34) |  | 2.11 | (1.89, 2.35) | | 1.51 | (1.32, 1.72) | |
| Regionalised, lymph nodes | 3.16 | (1.81, 5.51) |  | 5.31 | (4.37, 6.44) | | 3.59 | (2.75, 4.70) |  |
| Metastasised | 16.56 | (14.45, 18.99) |  | 7.42 | (6.82, 8.07) | | 3.04 | (2.52, 3.67) | |
| Unknown | 1.48 | (1.27, 1.72) |  | 1.28 | (1.19, 1.37) | | 1.12 | (1.04, 1.22) | |
| **Relationship status** |  |  | <0.001 |  |  | <0.001 |  |  | <0.001 |
| Married/partnered | Reference |  |  | Reference |  |  | Reference |  |  |
| Single | 1.29 | (1.15, 1.43) |  | 1.35 | (1.26, 1.45) | | 1.30 | (1.19, 1.42) | |
| Missing data | 0.74 | (0.36, 1.51) |  | 0.87 | (0.51, 1.50) |  | 1.16 | (0.49, 2.73) | |
| **Distance from nearest treatment facility** |  |  | 0.108 |  |  | 0.031 |  |  | 0.003 |
| <50 km | Reference |  |  | Reference |  |  | Reference |  |  |
| 50-99 km | 1.11 | (0.85, 1.44) |  | 1.10 | (0.95, 1.29) | | 1.24 | (1.03, 1.51) | |
| 100-149 km | 0.99 | (0.72, 1.36) |  | 1.13 | (0.94, 1.36) | | 1.18 | (0.94, 1.49) | |
| 150-199 km | 0.86 | (0.57, 1.31) |  | 1.19 | (0.94, 1.50) |  | 1.16 | (0.86, 1.57) | |
| 200+ km | 1.40 | (1.02, 1.93) |  | 1.38 | (1.14, 1.67) | | 1.62 | (1.27, 2.07) | |
| **Distance from nearest robotic surgery centre** |  |  | 0.289 |  |  | 0.024 |  |  | 0.572 |
| <50 km | Reference |  |  | Reference |  |  | Reference |  |  |
| 50-99 km | 0.88 | (0.66, 1.19) |  | 1.03 | (0.87, 1.22) | | 1.11 | (0.90, 1.37) |  |
| 100-149 km | 0.92 | (0.66, 1.27) |  | 0.98 | (0.81, 1.18) | | 1.10 | (0.86, 1.39) | |
| 150-199 km | 1.07 | (0.80, 1.43) |  | 0.91 | (0.76, 1.09) | | 1.06 | (0.85, 1.32) | |
| 200+ km | 0.82 | (0.66, 1.02) |  | 0.83 | (0.73, 0.95) | | 0.96 | (0.81, 1.12) | |
| **Health insurance status** |  |  | <0.001 |  |  | <0.001 |  |  | <0.001 |
| Private health insurance | Reference |  |  | Reference |  |  | Reference |  |  |
| No private insurance | 1.38 | (1.24, 1.53) |  | 1.32 | (1.24, 1.40) |  | 1.17 | (1.08, 1.26) | |
| Unknown/missing | 1.91 | (1.24, 2.94) |  | 0.67 | (0.42, 1.09) | | 0.27 | (0.11, 0.66) | |
| **Charlson comorbidity index (Quan)** |  |  | <0.001 |  |  | <0.001 |  |  | <0.001 |
| 0 | Reference |  |  | Reference |  |  | Reference |  |  |
| 1 | 1.32 | (1.12, 1.55) |  | 1.58 | (1.44, 1.73) | | 1.68 | (1.48, 1.89) | |
| 2 or more | 1.83 | (1.63, 2.04) |  | 2.40 | (2.25, 2.56) | | 4.18 | (3.86, 4.53) | |
| **Radical prostatectomy** |  |  | <0.001 |  |  | <0.001 |  |  | <0.001 |
| Yes | 0.14 | (0.10, 0.19) |  | 0.23 | (0.20, 0.26) |  | 0.45 | (0.40, 0.51) |  |
| No | Reference |  |  | Reference |  |  | Reference |  |  |
| **Radiation Therapy** |  |  | <0.001 |  |  | 0.003 |  |  | <0.001 |
| Yes | 0.45 | (0.40, 0.50) |  | 1.10 | (1.03, 1.17) | | 1.33 | (1.24, 1.44) | |
| No | Reference |  |  | Reference |  |  | Reference |  |  |

**Supplementary model 4 – Cox regression on cancer specific mortality, time varying analysis**

|  | 0-1 year | | | 1-5 years | | | 5+ years | | | | |  |
| --- | --- | --- | --- | --- | --- | --- | --- | --- | --- | --- | --- | --- |
| **Sociodemographic or health factor** | HR | 95% CI | p-value | HR | 95% CI | p-value | HR | | 95% CI | p-value | |  |
| **Aboriginal and Torres Strait Islander Identification** |  |  | 0.400 |  |  | 0.906 |  | |  | | 0.369 |  |
| Aboriginal and Torres Strait Islander | 1.21 | (0.77, 1.9) |  | 1.02 | (0.74, 1.4) |  | 1.26 | | (0.76, 2.08) | | |  |
| Non-Indigenous | Reference |  |  | Reference |  |  | Reference | |  | |  |  |
| **Age at diagnosis (years)** |  |  | <0.001 |  |  | <0.001 |  | |  | | <0.001 |  |
| <60 | Reference |  |  | Reference |  |  | Reference | |  | |  |  |
| 60-69 | 1.14 | (0.79, 1.64) |  | 1.13 | (0.94, 1.36) |  | 1.35 | | (1.01, 1.8) | |  |  |
| 70-79 | 2.05 | (1.45, 2.9) |  | 1.62 | (1.36, 1.93) |  | 2.19 | | (1.64, 2.92) | | |  |
| 80 or more | 5.37 | (3.83, 7.54) |  | 4.59 | (3.85, 5.49) |  | 7.14 | | (5.25, 9.73) | | |  |
| **Place of residence** |  |  | 0.008 |  |  | 0.001 |  | |  | | 0.061 |  |
| Major cities | Reference |  |  | Reference |  |  | Reference | |  | |  |  |
| Inner regional | 1.30 | (1.1, 1.54) |  | 1.25 | (1.11, 1.41) |  | 0.94 | | (0.75, 1.16) | | |  |
| Outer regional/remote/very remote | 1.41 | (1.02, 1.95) |  | 1.19 | (0.95, 1.48) |  | 0.62 | | (0.41, 0.93) | | |  |
| **Index of Relative Socioeconomic Disadvantage** |  |  | 0.660 |  |  | 0.326 |  | |  | | 0.926 |  |
| IRSD-1 (Most disadvantaged) | Reference |  |  | Reference |  |  | Reference | |  | |  |  |
| IRSD-2 | 1.08 | (0.89, 1.3) |  | 1.11 | (0.97, 1.26) |  | 1.10 | | (0.87, 1.39) | | |  |
| IRSD-3 | 0.92 | (0.75, 1.13) |  | 0.96 | (0.84, 1.11) |  | 1.04 | | (0.82, 1.33) | | |  |
| IRSD-4 | 1.01 | (0.82, 1.24) |  | 0.99 | (0.86, 1.15) |  | 1.02 | | (0.79, 1.3) | |  |  |
| IRSD-5 (Least disadvantaged) | 0.98 | (0.79, 1.22) |  | 1.01 | (0.87, 1.17) |  | 1.08 | | (0.84, 1.38) | | |  |
| **Year of Diagnosis** |  |  | 0.160 |  |  | <0.001 |  | |  | | <0.001 |  |
| 2009-2013 | 1.09 | (0.97, 1.23) |  | 1.46 | (1.34, 1.59) |  | 3.49 | | (2.42, 5.05) | | |  |
| 2014-2018 | Reference |  |  | Reference |  |  | Reference | |  | |  |  |
| **Degree of spread** |  |  | <0.001 |  |  | <0.001 |  | |  | | <0.001 |  |
| Localised | Reference |  |  | Reference |  |  | Reference | |  | |  |  |
| Regionalised, adjacent organs | 5.00 | (3.64, 6.88) |  | 3.13 | (2.66, 3.69) |  | 2.28 | | (1.79, 2.92) | | |  |
| Regionalised, lymph nodes | 5.76 | (2.8, 11.83) |  | 9.04 | (6.99, 11.69) |  | 7.05 | | (4.73, 10.51) | | |  |
| Metastasised | 39.42 | (31.95, 48.64) |  | 16.28 | (14.49, 18.29) |  | 8.95 | | (6.9, 11.61) | | |  |
| Unknown | 1.95 | (1.53, 2.49) |  | 1.69 | (1.51, 1.9) |  | 1.31 | | (1.1, 1.55) | |  |  |
| **Relationship status** |  |  | 0.003 |  |  | <0.001 |  | |  | | 0.069 |  |
| Married/partnered | Reference |  |  | Reference |  |  | Reference | |  | |  |  |
| Single | 1.26 | (1.1, 1.44) |  | 1.29 | (1.17, 1.43) |  | 1.24 | | (1.03, 1.49) | | |  |
| Missing data | 0.82 | (0.35, 1.92) |  | 0.58 | (0.24, 1.38) |  | 0.68 | | (0.09, 4.85) | | |  |
| **Distance from nearest treatment facility** |  |  | 0.104 |  |  | 0.378 |  | |  | | 0.001 |  |
| <50 km | Reference |  |  | Reference |  |  | Reference | |  | |  |  |
| 50-99 km | 1.26 | (0.91, 1.72) |  | 1.05 | (0.84, 1.31) |  | 1.33 | | (0.9, 1.95) | | |  |
| 100-149 km | 0.79 | (0.53, 1.19) |  | 1.19 | (0.93, 1.54) |  | 1.06 | | (0.65, 1.72) | | |  |
| 150-199 km | 0.92 | (0.55, 1.54) |  | 1.27 | (0.91, 1.76) |  | 1.31 | | (0.69, 2.49) | | |  |
| 200+ km | 1.27 | (0.85, 1.91) |  | 1.26 | (0.95, 1.69) |  | 2.67 | | (1.63, 4.37) | | |  |
| **Distance from nearest robotic surgery centre** |  |  | 0.083 |  |  | 0.575 |  | |  | | 0.121 |  |
| <50 km | Reference |  |  | Reference |  |  | Reference | |  | |  |  |
| 50-99 km | 0.81 | (0.56, 1.16) |  | 1.06 | (0.83, 1.36) |  | 1.42 | | (0.93, 2.17) | | |  |
| 100-149 km | 0.97 | (0.66, 1.45) |  | 0.94 | (0.72, 1.24) |  | 1.68 | | (1.04, 2.71) | | |  |
| 150-199 km | 1.02 | (0.71, 1.45) |  | 0.93 | (0.72, 1.2) |  | 0.94 | | (0.57, 1.55) | | |  |
| 200+ km | 0.71 | (0.54, 0.93) |  | 0.89 | (0.74, 1.07) |  | 1.13 | | (0.82, 1.58) | | |  |
| **Health insurance status** |  |  | <0.001 |  |  | <0.001 |  | |  | | 0.056 |  |
| Private health insurance | Reference |  |  | Reference |  |  | Reference | |  | |  |  |
| No private insurance | 1.30 | (1.14, 1.48) |  | 1.27 | (1.16, 1.39) |  | 1.21 | | (1.04, 1.42) | | |  |
| Unknown/missing | 1.73 | (1.01, 2.98) |  | 0.48 | (0.22, 1.02) |  | - | - | | | |  |
| **Charlson comorbidity index (Quan)** |  |  | 0.001 |  |  | <0.001 |  | |  | | <0.001 |  |
| 0 | Reference |  |  | Reference |  |  | Reference | |  | |  |  |
